# A 4-week high-AGE diet does not impair glucose metabolism and vascular function in obese individuals

**DOI:** 10.1172/jci.insight.156950

**Published:** 2022-03-22

**Authors:** Armand M.A. Linkens, Alfons J.H.M. Houben, Petra M. Niessen, Nicole E.G. Wijckmans, Erica E.C. de Goei, Mathias D.G. Van den Eynde, Jean L.J.M. Scheijen, Marjo P.H. van den Waarenburg, Andrea Mari, Tos T.J.M. Berendschot, Lukas Streese, Henner Hanssen, Martien C.J.M. van Dongen, Christel C.J.A.W. van Gool, Coen D.A. Stehouwer, Simone J.M.P. Eussen, Casper G. Schalkwijk

**Affiliations:** 1 Department of Internal Medicine, Maastricht University Medical Center, Maastricht, Netherlands.; 2 CARIM School for Cardiovascular Diseases,; 3 Department of Epidemiology, and; 4 CAPHRI School for Care and Public Health Research Unit, Maastricht University, Maastricht, Netherlands.; 5 Institute of Neuroscience, National Research Council, Padua, Italy.; 6 University Eye Clinic Maastricht, Maastricht University Medical Center, Maastricht, Netherlands.; 7 Division of Sports and Exercise Medicine, Department of Sport, Exercise and Health, University of Basel, Basel, Switzerland.

**Keywords:** Clinical Trials, Vascular Biology, Glucose metabolism, Microcirculation

## Abstract

**BACKGROUND:**

Accumulation of advanced glycation endproducts (AGEs) may contribute to the pathophysiology of type 2 diabetes and its vascular complications. AGEs are widely present in food, but whether restricting AGE intake improves risk factors for type 2 diabetes and vascular dysfunction is controversial.

**METHODS:**

Abdominally obese but otherwise healthy individuals were randomly assigned to a specifically designed 4-week diet low or high in AGEs in a double-blind, parallel design. Insulin sensitivity, secretion, and clearance were assessed by a combined hyperinsulinemic-euglycemic and hyperglycemic clamp. Micro- and macrovascular function, inflammation, and lipid profiles were assessed by state-of-the-art in vivo measurements and biomarkers. Specific urinary and plasma AGEs N^ε^-(carboxymethyl)lysine (CML), N^ε^-(1-carboxyethyl)lysine (CEL), and N^δ^-(5-hydro-5-methyl-4-imidazolon-2-yl)-ornithine (MG-H1) were assessed by mass spectrometry.

**RESULTS:**

In 73 individuals (22 males, mean ± SD age and BMI 52 ± 14 years, 30.6 ± 4.0 kg/m^2^), intake of CML, CEL, and MG-H1 differed 2.7-, 5.3-, and 3.7-fold between the low- and high-AGE diets, leading to corresponding changes of these AGEs in urine and plasma. Despite this, there was no difference in insulin sensitivity, secretion, or clearance; micro- and macrovascular function; overall inflammation; or lipid profile between the low and high dietary AGE groups (for all treatment effects, *P* > 0.05).

**CONCLUSION:**

This comprehensive RCT demonstrates very limited biological consequences of a 4-week diet low or high in AGEs in abdominally obese individuals.

**TRIAL REGISTRATION:**

Clinicaltrials.gov, NCT03866343; trialregister.nl, NTR7594.

**FUNDING:**

Diabetesfonds and ZonMw.

## Graphical abstract

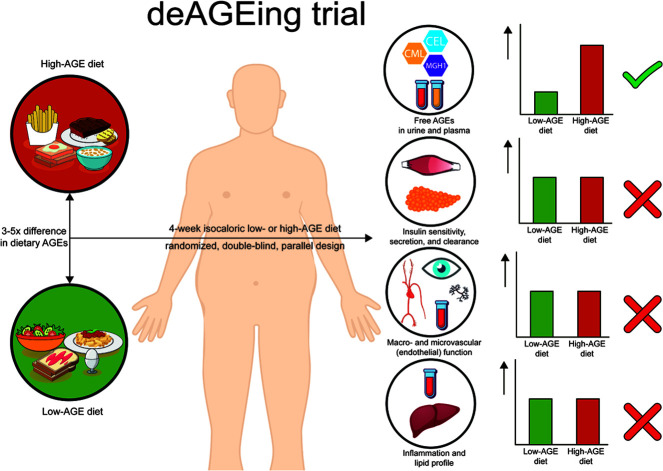

## Introduction

In vivo accumulation of advanced glycation endproducts (AGEs), a heterogeneous group of sugar-modified amino groups within proteins and other macromolecules, may drive the pathophysiology of type 2 diabetes (1–6) and its associated vascular dysfunction (7–11). In addition to the endogenous formation of AGEs, AGEs also form in foods, especially those rich in sugar and protein or fat, when exposed to heat (12). Since heating of food is widely employed due to favorable effects on sterility, flavor, and color, diets consumed in Westernized societies significantly contribute to the body’s exposure to AGEs (13). Studies in animals (14, 15) and humans (13) suggest that these dietary AGEs are absorbed and that a diet high in AGEs may induce endothelial dysfunction and inflammation (16, 17), insulin resistance (18), and β cell dysfunction (2). However, whether reducing AGE intake improves risk factors for type 2 diabetes and vascular dysfunction remains controversial (19).

Studies so far mainly employed different cooking techniques to modulate dietary AGEs, such as boiling versus frying, or did not match intervention and control diets for calories and macronutrients (20–24). Additionally, dietary AGEs were almost exclusively estimated using an IHC-based database (25) and not with the current gold standard instrumental method ultraperformance liquid chromatography–tandem mass spectrometry (UPLC-MS/MS) (26–28). Observations from the above-mentioned studies (2, 16–18) may be attributed to factors other than dietary AGEs. Limitations from these previous studies were partly circumvented in a recent crossover randomized controlled trial (RCT) in obese individuals, where insulin sensitivity improved after a 2-week low- compared with a high-AGE diet (29). However, several important knowledge gaps remain. Although dietary AGEs did not influence insulin secretion in response to an i.v. glucose bolus, this was not determined by C-peptide deconvolution and was not adjusted for the observed change in insulin sensitivity. Additionally, insulin clearance, increasingly recognized as an independent risk factor for type 2 diabetes (30), was not investigated in this study. Furthermore, micro- and macrovascular function, important contributors to insulin sensitivity (31), β cell function (32, 33), and cardiovascular risk (34–36) have not yet been investigated in a well-controlled RCT.

The aim of the present study was to investigate the effects of dietary AGEs on factors involved in the pathogenesis of type 2 diabetes and on vascular function. To this end, we investigated — in a double-blind, parallel RCT — the effects of 4-week isocaloric and macronutrient-matched diet either low or high in AGEs on insulin sensitivity, secretion, and clearance; micro- and macrovascular function; inflammatory markers; and lipid profile in abdominally obese individuals using state-of-the-art methods.

## Results

### Baseline characteristics.

**Figure 1:**
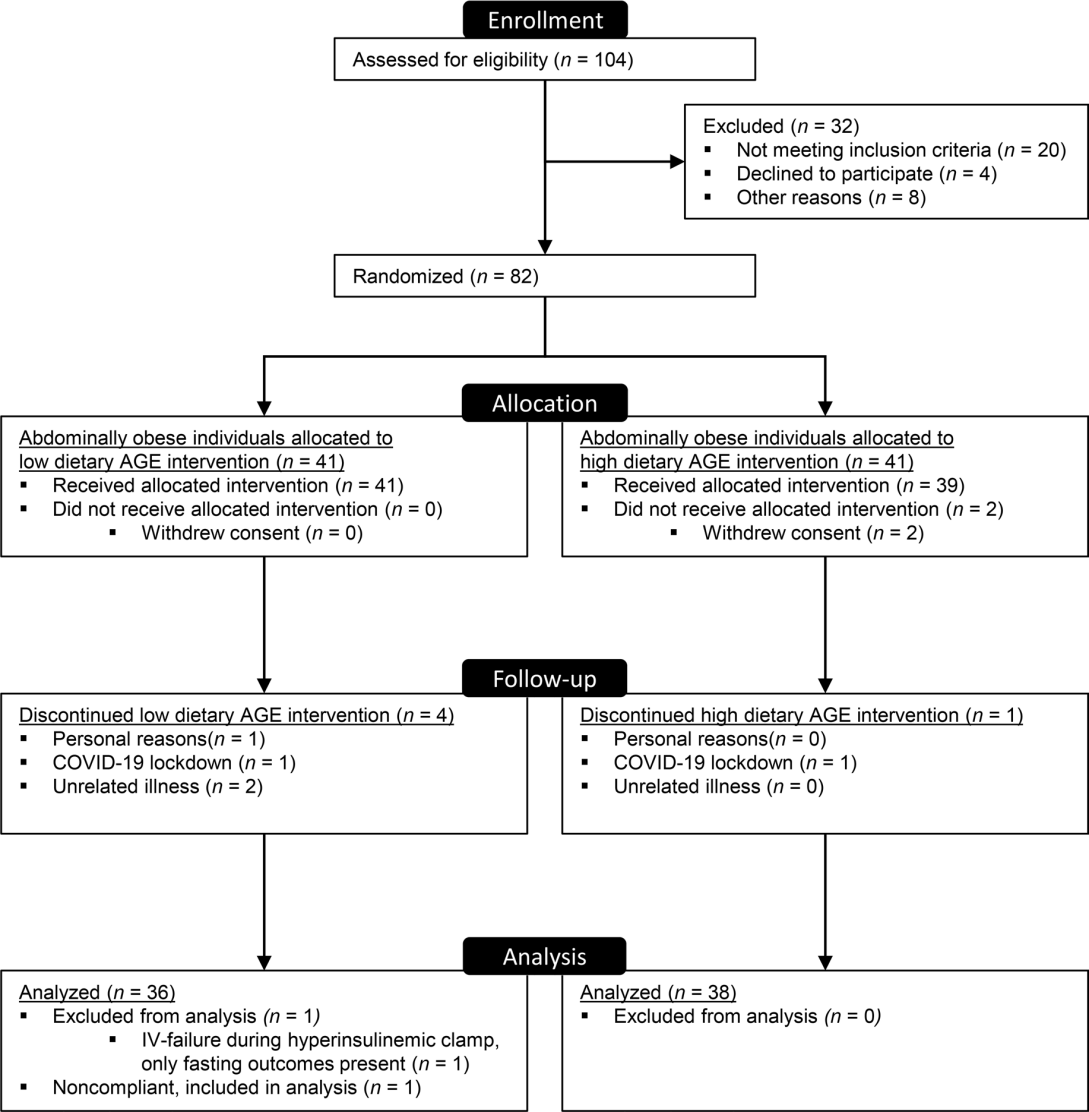
Consort flowchart.

Of 82 enrolled participants, insulin sensitivity was available in 73 participants (Figure 1). Dropouts occurred unrelated to the dietary intervention, and characteristics of these participants were largely comparable with those included in the complete case analysis (Supplemental Table 1; supplemental material available online with this article; https://doi.org/10.1172/jci.insight.156950DS1). By design, all participants were abdominally obese, nonsmokers, and free of apparent cardiovascular disease (Table 1). We included more females (*n =* 51) than males (*n =* 22) by chance. Baseline characteristics were not different between groups, except for habitual intake of dietary N^ε^-(carboxymethyl)lysine (CML) and N^ε^-(1-carboxyethyl)lysine (CEL), which was higher in the low-AGE group compared with the high-AGE group (age, sex, and energy-intake adjusted *P =* 0.007 for CML, *P =* 0.004 for CEL, and *P =* 0.08 for N^δ^-[5-hydro-5-methyl-4-imidazolon-2-yl]-ornithine [MG-H1]). This was attributable to higher consumption of several high-AGE foods by participants in the low-AGE group (beef stew, peanuts, peanut butter, and chocolate milk).

### Dietary intake during the intervention.

The intake of dietary AGEs CML, CEL, and MG-H1, assessed from two 5-day food diaries at weeks 1 and 4 of the dietary intervention, were markedly different during the low- and high-AGE diets, with daily intake for CML of 2.63 ± 0.68 mg/day versus 7.02 ± 1.60 mg/day, for CEL of 1.69 ± 0.40 mg/day versus 9.03 ± 2.23 mg/day, and for MG-H1 of 13.44 ± 3.15 mg/day versus 49.67 ± 13.92 mg/day (Table 2). This was also confirmed by 24-hour recalls in weeks 2 and 3 of the dietary intervention (Supplemental Table 2). Compared with habitual intake assessed by a food frequency questionnaire (FFQ), all participants showed a decreased intake in at least 1 of 3 dietary AGEs during the low-AGE diet and increased intake in at least 1 of 3 dietary AGEs during the high-AGE diet (Supplemental Figure 1). We also determined the habitual intake of dicarbonyls, highly reactive compounds that give rise to rapid formation of AGEs. The intake of these dietary dicarbonyls were also different during both diets, although less marked (Table 2).

Energy intake during the intervention and body weight after the intervention were not statistically different between groups (Tables 2 and 3). Although the intervention diets were designed to be macronutrient matched, intake of energy as fat was slightly lower in favor of carbohydrates during the low-AGE diet: mean energy percentage ± SD of 31.5% ± 2.6% versus 35.8% ± 3.1%, *P* < 0.001 for fat; and 48.4% ± 2.6% versus 44.5% ± 2.8%, *P* < 0.001 for carbohydrates (Supplemental Figure 2). Intake of fiber was marginally but statistically higher during the low-AGE diet compared with the high-AGE diet: mean energy percentage ± SD of 2.1% ± 0.2% versus 2.3% ± 0.1%, *P* < 0.001. Consumption of alcohol was not statistically different between both groups (data not shown). There also were some differences in micronutrient intake (Supplemental Table 3). No diet-related adverse effects were reported.

### AGEs and dicarbonyls in plasma, urine, and skin.

**Figure 2:**
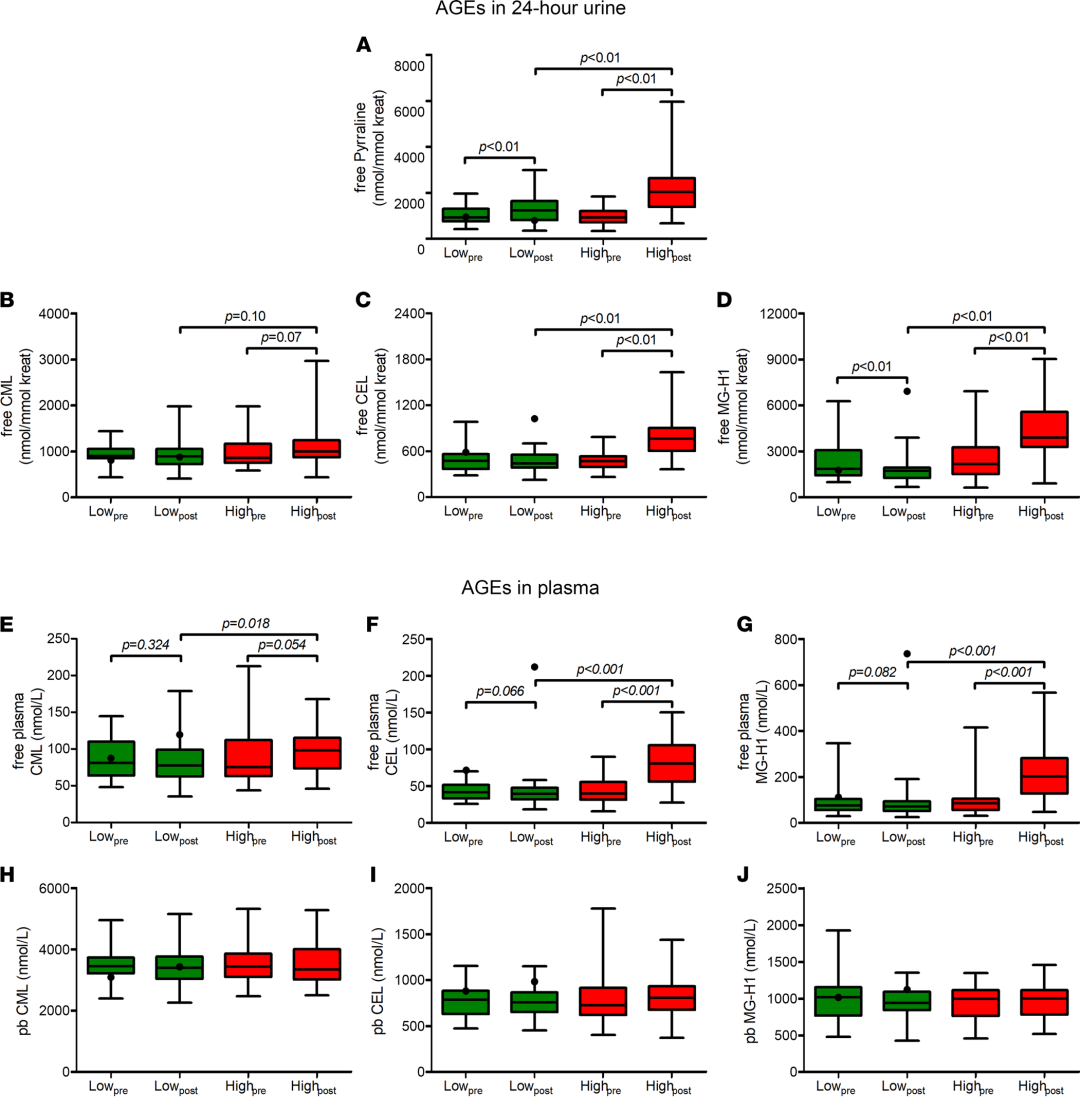
Box-and-whisker plots of AGEs in 24-hour urine and plasma before and after a 4-week low- or high-AGE diet in abdominally obese individuals. (**A**–**J**) Black lines indicate median, box edges first and third quartiles, and whiskers indicate minimum and maximum of all data. One participant was deemed noncompliant and was not included in statistical analyses of these variables. This participant is shown as a black dot. Differences within groups after the intervention were assessed by a paired-samples *t* test, whereas differences between groups after the intervention period were assessed by 1-way ANCOVA with sex, age, and the baseline value of the outcome of interest as a covariate. *n* = 36 and *n* = 38 for low- and high-AGE diets, respectively. CEL, N^ε^-(1-carboxyethyl)lysine; CML, Nε-(carboxymethyl)lysine; MG-H1, N^δ^-(5-hydro-5-methyl-4-imidazolon-2-yl)-ornithine; pb, protein-bound.

In line with their intake, levels of free AGEs CML, CEL, and MG-H1 in plasma and 24-hour urine were higher after the high-AGE diet as compared with low-AGE diet (Table 3, Supplemental Table 4, and Figure 2), although the difference for free CML in urine did not reach statistical significance (*P =* 0.07). The AGE pyrraline, suggested to be derived mainly from food intake and not from endogenous formation (37), also showed increased levels in urine after the high- compared with low-AGE diet. Protein-bound AGEs in plasma were not statistically different after the low- compared with high-AGE diet (Table 3, Supplemental Table 4, Figure 2). Skin autofluorescence, an estimate of fluorescent AGE accumulation in skin (38), was also not statistically different after the low- compared with high-AGE diet (Supplemental Table 3). Of note, 1 participant allocated to the low-AGE diet showed a profound increase in urinary and plasma levels of free CEL and MG-H1 after the intervention diet (Figure 2, black dots). This participant was deemed noncompliant and was not included in the analyses regarding AGEs in plasma and urine. In line with our intention-to-treat design, this participant was included in all other analyses. Exclusion of this participant did not materially change all other outcomes (data not shown).

Levels of AGEs in 24-hour urine correlated with their corresponding average daily intake during the intervention (Pearson’s *r* = 0.26 [*P =* 0.03] for CML, *r* = 0.57 [*P* < 0.001 for CEL], and *r* = 0.61 [*P* < 0.001] for MG-H1). Although intake of dicarbonyls was also slightly higher during the high-AGE diet, we observed no difference in their levels in plasma and 24-hour urine after both diets (Supplemental Table 3).

### Glucose metabolism.

Fasting indices of glucose metabolism (plasma glucose, plasma insulin, plasma C-peptide, insulin secretion, and insulin clearance) were not statistically different after the low- and high-AGE diets (Supplemental Table 5).

Insulin sensitivity was not statistically different after the low- compared with the high-AGE diet, with overall difference of –0.5 mg/kg/min (95% CI, –1.3 to 0.2), representing a ~10% nonsignificant change (Table 3). Additional adjustment for plasma insulin (M/I; insulin sensitivity divided by mean plasma insulin concentration during the 90- to 120-minute timeframe of the hyperinsulinemic clamp) did not materially change these findings (data not shown). Likewise, insulin clearance and C-peptide suppression (reflecting insulin’s potential to inhibit its own secretion; ref. 39) were not statistically different after both diets (Table 3 and Supplemental Table 5).

Finally, we determined insulin secretion indices during the hyperglycemic clamp. Following the i.v. glucose bolus, plasma glucose was sharply raised by the desired increment of 2.80 mmol/L (Supplemental Figure 3). The low- or high-AGE diet had no effect on the corresponding increase in first-phase insulin secretion rate (ISR), with an overall difference of 179 pmol/min/m^2^ (95% CI, –19 to 52) (Table 3). Adjusting for the variability in glucose increment by adding it as a variable in the ANCOVA model did not materially change these findings (data not shown). After the glucose bolus, plasma glucose was maintained at the 2.80 mmol/L increment by a variable glucose infusion. The resulting second-phase ISR during the remaining 20 minutes of the hyperglycemic clamp, as well as the last 10 minutes only (at which plasma glucose was more stable), were not statistically different after the low- compared with high-AGE diet (Table 3 and Supplemental Table 5). β Cell glucose sensitivity was also not different after the low- compared with the high-AGE diet (Supplemental Table 5). Additionally adjusting these indices for insulin sensitivity did not materially change these findings (data not shown).

### Microvascular function.

Insulin-mediated microvascular recruitment (IMMR) in skeletal muscle and the skin microvascular response to local heating, reflecting in vivo skeletal muscle and skin microvascular function, were not statistically different after the low- compared with the high-AGE diet; the overall difference for IMMR was –3.1% (95% CI, –19.5 to 13.4) (Table 3 and Supplemental Table 5). Likewise, microvascular retinal vessel calibers, skin microvascular flowmotion, the *Z* score of endothelial dysfunction plasma biomarkers, or the individual markers were also not statistically different between the low- compared with the high-AGE diet (Table 3 and Supplemental Table 6).

### Macrovascular function.

Macrovascular endothelial function, assessed by flow-mediated dilation, was not statistically different after the low- compared with the high-AGE diet: the overall difference was –0.1% (95% CI, –1.5 to 1.2) (Table 3). Aortic stiffness, assessed by carotid-femoral pulse wave velocity (cfPWV); carotid stiffness, assessed by carotid distensibility coefficient (DC) and carotid Young’s elastic modulus (YEM); carotid intima-media thickness (IMT); augmentation index; and 24-hour systolic and diastolic blood pressure were not statistically different following both diets (Supplemental Table 6). Additional adjustment of the macrovascular analyses for systolic blood pressure did not materially change the results (data not shown).

### Inflammatory markers.

With all the plasma inflammatory markers combined in a *Z* score, there was no statistically significant difference after the low- compared with the high-AGE diet, with overall difference of 0.18 SD (95% CI, –0.08 to 0.44) (Table 3). Of the individual plasma inflammatory markers, only adiponectin decreased after the low-AGE diet, which resulted in an overall difference of –1.54 μg/mL (95% CI, –2.37 to –0.71) after the low- compared with the high-AGE diet (Supplemental Table 7).

Absolute monocyte count was reduced after the high-AGE diet, which resulted in overall difference of 0.05 × 10^9^/L (95% CI, 0.01 to 0.08) after the low- compared with the high-AGE diet (Supplemental Table 7). Counts of leucocytes, segmented granulocytes, lymphocytes, eosinophils, and basophils were not statistically different after the low- compared with the high-AGE diet.

### Cholesterol and liver markers.

Triglycerides, LDL and HDL cholesterol, γ-glutamyl transferase (γ-GT), and the fatty liver index were not statistically different after the low- compared with the high-AGE diet (Supplemental Table 8).

### Markers of DNA glycation.

*N^2^*-(1-carboxyethyl)-2’-deoxyguanosine (CEdG) levels in 24-hour urine, reflecting DNA glycation, decreased after the low-AGE diet and increased after the high-AGE diet, resulting in a statistically significant overall increase after the high- compared with low-AGE diet (Table 3). 8-Oxo-2’-deoxyguanosine (8-Oxo-dG) levels in 24-hour urine, reflecting DNA oxidation, were not statistically different after the low- compared with the high-AGE diet.

### Sensitivity analyses.

The increase in plasma adiponectin and urinary CEdG and the decrease in absolute monocyte count after the low- compared with high-AGE diet remained statistically significant, with further adjustment of the ANCOVA model for carbohydrate, protein, and fat intake (data not shown). To further assess the robustness of these findings, we also performed multiple linear regression to determine associations between indices of AGE intake and these outcomes. While adjusting for age, sex, and intake of carbohydrate, protein, and fat, daily intake of CML, CEL, and MG-H1 were positively associated with plasma adiponectin, while levels of free CML in urine were inversely associated with plasma adiponectin (Supplemental Table 9). There was a trend for an inverse association between free plasma CML and absolute monocyte count (*P =* 0.05), while intake of all AGEs and all free AGEs in plasma and urine were positively associated with urinary CEdG (Supplemental Table 9).

Interestingly, with additional adjustment for macronutrient intake in the ANCOVA model, some additional outcomes became statistically different after the low- compared with the high-AGE diet. cfPWV and serum HDL were lower, while serum triglycerides were higher after the low- compared with the high-AGE diet, with an overall difference of –0.78 m/s (95% CI, –1.53 to –0.02), –0.10 mmol/L (95% CI, –0.19 to –0.01), and 0.23 mmol/L (95% CI, 0.05 to 0.40), respectively (data not shown). However, only for serum HDL and triglycerides, and not for cfPWV, we found statistically significant associations with indices of dietary AGE intake. Intake of CML, CEL, and MG-H1 were positively associated with serum HDL (Supplemental Table 9). Intake of CML, CEL, and MG-H1, and free CEL and MG-H1 in plasma and urine, were inversely associated with serum triglycerides (Supplemental Table 9).

Additionally, adjusting for magnesium and selenium intake — micronutrients associated with insulin sensitivity — did not materially change our findings (data not shown). Of all other outcomes, only serum HDL become lower after the low- compared with the high-AGE diet after additional adjustment for selenium intake (data not shown).

## Discussion

Here, we present short-term biological effects of dietary AGEs using state-of-the-art methods. Despite a marked difference in intake of dietary AGEs, a 4-week diet low in AGEs compared with one high in AGEs did not change insulin sensitivity, secretion, and clearance; vascular function; or overall inflammation of healthy but abdominally obese individuals.

We supplied a profound difference in AGE intake between diets — 167% for CML, 434% for CEL, and 270% for MG-H1 — obtained without drastically altering food preparation methods and with similar energy content. Importantly, the comparison with habitual AGE intake revealed that, indeed, all participants lowered or increased their intake in line with their treatment allocation. Compliance was further confirmed by the increased levels of free AGEs in 24-hour urine and plasma after the high- compared with the low-AGE diet. Despite this, we observed no difference in insulin sensitivity after both diets. In contrast, De Courten et al. showed an improvement in insulin sensitivity by 2.1 mg/kg/min after a 2-week low compared with high-AGE diet in a well-controlled RCT with crossover design (29). Since AGE intake was estimated in both studies using the same UPLC-MS/MS dietary AGE database, they are directly comparable. Interestingly, AGE intake during our low-AGE diet was lower, while it was also higher during our high-AGE diet when compared with the corresponding diets by De Courten et al. (29). Other differences that may have contributed to our inconsistent findings are the crossover design, study population, and design of the dietary intervention. Although crossover bias was statistically excluded in their analyses, an incomplete washout could still have contributed to the change in insulin sensitivity observed after the second intervention period. Additionally, they included slightly younger and more insulin-sensitive participants (age 34 ± 10 years, M-value 7.0 ± 2.5 mg/kg/min), and effects of low- and high-AGE diet might be more profound in this group when compared with our older and less insulin-sensitive participants. Importantly, differences in AGE content of low- and high-AGE diets in the De Courten study were achieved by carefully matching low- and high-AGE food products based on differences in cooking techniques (40). In contrast, our low- and high-AGE diets were designed as stand-alone diets, and differences in AGEs were not solely achieved by cooking methods. Although this may explain why we also found more differences in micronutrients between our diets, our approach reduces the possibility that large differences in food preparation methods have confounded our results. Furthermore, it is unlikely that the small difference in fat content between our low- and high-AGE diets masked a change in insulin sensitivity, since far greater differences were needed in intervention trials to elicit such an effect (41), and we also found no difference in insulin sensitivity while adjusting for macronutrient intake. Another possibility is that an acute dietary AGE-induced change in insulin sensitivity is not sustained after 2 weeks. However, in line with the unchanged insulin sensitivity in the present study, all other outcomes of glucose metabolism also showed no change. The first-phase insulin secretion response, reduced in individuals with impaired glucose tolerance (42), was not changed after the low- or high-AGE diet. This was also reported by De Courten et al. (29). We extend on these findings by also investigating the second-phase insulin response, β cell glucose sensitivity, and fasting insulin secretion, which were also all unaffected by the low- or high-AGE diet. Importantly, our indices of β cell function were determined by the gold standard hyperglycemic clamp. Furthermore, these findings were independent of hepatic insulin clearance and insulin sensitivity, as we determined ISRs by C-peptide deconvolution (43), which is not affected by hepatic insulin clearance, and adjusted our final analyses for insulin sensitivity. Furthermore, we are the first to our knowledge to investigate the effects of dietary AGEs on insulin clearance, which is increasingly recognized as an important determinant of type 2 diabetes risk (30, 44) and was recently shown to predict type 2 diabetes in Native Americans, independently of insulin sensitivity and secretion (30). However, we observed no difference in insulin clearance during the fasting state, as well as during hyperinsulinemia after the low- or high-AGE diet.

Despite the increase of free AGEs in plasma, indicating higher exposure of the vascular endothelium to AGEs, we observed no deterioration of an extensive panel of macrovascular and microvascular function measurements. Ultimately, this is not surprising, since we did also not observe supporting differences in the potential underlying pathophysiological mechanisms (24-hour blood pressure, insulin sensitivity, lipid profile, inflammatory markers, plasma biomarkers of endothelial dysfunction, and oxidative stress). If anything, there was a slight improvement in inflammation and lipid profile after the high-AGE diet, as is apparent from the decrease in total monocyte count and serum triglycerides and from the increase in adiponectin and serum HDL. The lower cfPWV observed after the low- compared with the high-AGE diet occurred only when additionally adjusting for macronutrient intake, and it is likely a chance finding. In contrast to all other outcomes, we did not observe an association between any of the markers of dietary AGE intake and cfPWV. Overall, these findings are in line with those of our 2 previous observational studies in the population-based cohort of The Maastricht Study, where we observed no association between habitual intake of dietary AGEs and both arterial stiffness (45) and generalized microvascular function (46). These results are seemingly in contrast with the role of endogenously formed AGEs in microvascular dysfunction (47, 48) and arterial stiffening (49–51). Specifically, AGEs have been linked to arterial stiffness and microvascular dysfunction in several studies (47, 48) via mechanisms that include collagen crosslinking within the vascular wall (51) and endothelial dysfunction via stimulation of the receptor for AGEs (RAGE) (52, 53), However, as collagen crosslinking occurs during the formation of AGEs in the vascular wall, and dietary AGEs did not show affinity for RAGE (54), both mechanisms are unlikely to apply to AGEs of dietary origin. Combined, this suggests a limited role of dietary AGEs in micro- and macrovascular function in humans.

Interestingly, we did observe increased levels of CEdG in urine after the high-AGE diet, reflecting DNA glycation by methylglyoxal (MGO) (55). Formation of CEdG by MGO is considered highly mutagenic, as it is accompanied by guanine transversions and base deletions (56) and by single-strand DNA breaks (57). The finding of increased CEdG after the high-AGE diet provides an interesting topic of future research, given the increased risk of several cancer types with higher habitual AGE intake (58–61).

Our broad array of null findings are not in agreement with those of previous animal and human studies. In mice, baked chow diets high in AGEs led to impaired insulin secretion (2), insulin resistance and T2DM (62), and arterial stiffness (63). However, the usage of baked chow diets may have led to other effects than solely increasing dietary AGEs, such as decreased vitamin bioavailability and increased acrylamide formation. Additionally, AGE levels in baked chow may be higher than those in human food. The relevance of these findings in mice for humans seems limited. Data in humans are less consistent, but metaanalyses of RCTs suggest that a high-AGE diet leads to insulin resistance (64), inflammation, endothelial dysfunction (16), and atherogenic dyslipidemia (17). However, most RCTs in these metaanalyses are deemed of low methodological quality (18, 19, 65). Additionally, dietary AGEs were mostly modulated by employing largely different food preparation methods (i.e., steaming/boiling versus grilling/frying). The limitations of using baked diets described above also applies to these human studies. Furthermore, most intervention diets were not matched for energy content or energy intake was not reported (21, 22, 24). Effects observed in these studies may not be attributed to dietary AGEs alone. Importantly, AGEs were measured in most studies using ELISA, which shows limited reliability compared with the gold standard UPLC-MS/MS. Thus, the true levels of AGEs in food, plasma, and urine in these studies are unknown. Another limitation was that, in most previous trials, both participants and investigators were not masked for treatment allocation (20–24, 66, 67). Therefore, current data from literature are insufficient to conclude that dietary AGEs pose a threat to human health (19).

The present study has several strengths. Mainly, we measured an extensive panel of outcomes concerning glucose metabolism, vascular function, and inflammation using state-of-the-art methods. Additionally, AGEs and dicarbonyls in food, plasma, and urine were determined using the gold-standard UPLC-MS/MS. Our intervention diets were specifically designed and not solely based on food preparation methods, making them directly translatable to daily practice. Compliance to the intervention diets was enhanced by frequent checkups by our trained dieticians and by delivering most food items to the participants free of charge. Compliance was confirmed by both food diaries, 24-hour recalls, and AGEs in plasma and urine. Furthermore, both the investigators and participants were blinded to the participant’s treatment allocation, and the investigators remained blinded until the statistical analyses were finalized. Although it is theoretically possible that participants discovered their allocation themselves, this is not evident, since the low-AGE diet also contained some fried and toasted foods.

The present study also has several potential limitations. Most importantly, because of the relatively short intervention duration of 4 weeks, we are unable to draw conclusions on longer-term effects of a diet low or high in AGEs. However, in line with the current data, we recently showed no associations between habitual intake of dietary AGEs, assessed by an FFQ with a reference period of 1 year, and arterial stiffness and generalized microvascular function in a population-based cohort (45, 46). Another limitation is that the habitual intake of AGEs, as assessed by an FFQ, was higher in our low-AGE group than in our high-AGE group; however, this occurred by chance, since almost all other outcomes showed no imbalance at baseline. This imbalance is expected to enhance the dietary effects, due to the greater differences with the habitual diet. Additionally, participants were abdominally obese but otherwise healthy White individuals from Western Europe, and extrapolating our findings to groups with other metabolic characteristics or individuals of other ethnicities should be done with caution. Specifically, whether short-term modulation of AGE intake influences the current outcomes in those with diabetes of impaired kidney function cannot be deduced from the current study. Also, we cannot exclude the possibility that disparities in micronutrients between diets may have confounded our results. However, regarding vitamins, the difference in daily intake between groups is far less than dosages used in intervention trials in which these vitamins were associated with health improvements (68, 69). Moreover, both intervention diets were constructed to contain the recommended daily requirements for all micronutrients. Furthermore, as dietary AGEs are derived from whole foods, a low- or high-AGE diet will always be accompanied by disparities in some nutrients. Also, we did not monitor physical activity during the intervention period. Although all participants were instructed not to alter their physical activity pattern, increased awareness of dietary habits during the intervention could lead to short-term changes in lifestyle. However, if this occurred, we have no reason to suspect why this would affect one intervention group more than the other. Lastly, although the intervention diets were designed to differ in AGEs, dicarbonyls — reactive precursors to AGEs (70) — were also lower in the low- compared with the high-AGE diet, albeit to a much lesser extent. We cannot exclude that dietary dicarbonyls contributed to the present findings. Due to their common source (the Maillard reaction), future studies reporting health effects of either dietary AGEs or dicarbonyls should consider this, as well.

In conclusion, we provide the most extensive overview to our knowledge that a 4-week diet low or high in AGEs has no effect on insulin sensitivity, secretion, or clearance; vascular function; or overall inflammation in abdominally obese but healthy individuals. These findings require validation in large prospective cohort studies and in populations with established disease such as diabetes and kidney failure.

## Methods

*Study design*. In this double-blind, parallel design, RCT (deAGEing trial; https://www.trialregister.nl/trial/7386) participants were assigned at a 1:1 ratio to a 4-week dietary intervention low or high in AGEs. Randomization was performed remotely from the recruitment center by an independent investigator to the research team and participants after email verification of the randomization criteria. The randomization sequence was generated by this independent researcher with an online randomization tool (randomization.com, original generator) using randomly permutated block sizes of 4, with stratification for age (below and above 50 years of age) and sex. Both the investigators and participants were formally blinded to the treatment allocation, and participants were instructed not to inform the investigators about the food items in their dietary intervention. The study dietician was aware of the treatment allocation.

*Study population*. Eighty-two abdominally obese but otherwise healthy individuals were recruited by advertisements and enrolled at the Maastricht University Medical Center, from November 2018 to March 2021. Eligible for inclusion were all individuals aged 18 and older and abdominally obese (waist circumference > 88 cm for females; waist circumference > 102 for males; ref. 71). Noneligible individuals were those with diabetes (fasting plasma glucose > 7.0 mmol/L, HbA1c > 6.5%, or self-reported use of glucose-lowering medication), CVD (medical history), a history of smoking (cessation < 1 year ago), hyperlipidemia (total cholesterol > 8 mmol/L, triglycerides > 4 mmol/L, or use of lipid-lowering medication), a history of using medication known to influence glucose metabolism or vascular function (e.g., glucocorticosteroids, NSAIDs), an inability to stop antihypertensive medication for 8 weeks, a current pregnancy, unstable body weight (change > 3 kg in the last 2 months), a history of the use of dietary supplements within the previous month, or significant food allergies or intolerance.

*Sample size calculation*. The primary objective was to determine a change in insulin sensitivity assessed by the hyperinsulinemic-euglycemic clamp. A previous cross-over design RCT with a comparable study population and intervention diet found an improvement of 1.3 ± 1.8 mg/kg/min in insulin sensitivity after a 2-week low-AGE diet relative to baseline (29). Due to our parallel design, we expected greater variance between participants. However, our intervention period was 4 weeks rather than 2, so the effect size was expected to be larger. We expected an improvement of insulin sensitivity of 1.5 ± 2.1 mg/kg/min. Using the PS Power and Sample Size Calculations Software program (version 3.1.2), based on a 2-tailed significance level of 0.05 and a power of 0.85, 36 individuals per group were needed to detect a statistical difference. Considering a drop-out rate of 12%, we included 41 participants per group, resulting in a total of 82 participants.

*Run-in diet*. Prior to the baseline measurement, all participants followed an isocaloric 2-day run-in dietary schedule. Participants’ habitual energy intake was assessed by a 3-day food diary, including 2 weekdays and 1 weekend day. The run-in diet contained an average amount of dietary AGEs, based on intake in a large population-based cohort (45), and it was designed to exclude any influences of high-AGE products consumed the days prior to the baseline measurement. Habitual intake of AGEs was assessed by a validated FFQ, as described in detail in the Supplemental Methods.

*Dietary intervention*. Intervention diets were constructed by a trained dietician and were energy- and macronutrient-matched. Both intervention diets adhered to the Dutch dietary guidelines for macro- and micronutrient intake (72) and contained 15% protein, 35% fat, 48% carbohydrates, and 2% fiber. With the use of our gold-standard UPLC-MS/MS dietary AGE database that contains approximately 250 food items (28), a theoretical difference of approximately 75% in dietary AGEs was attained between diets. Participants prepared their food at home using predefined recurring weekly menus. Most food items were provided to the participants free of charge by means of a delivery service. Participants were instructed not to change their habitual portion sizes or habitual clock times of food intake, not to attempt changes in body weight, and not to consume food supplements during the duration of the study.

*Compliance*. Adherence to the dietary intervention was measured in 3 ways. First, participants kept a 5-day dietary record in the first and last week of the intervention period. Second, participants were additionally contacted in the second and third week of the intervention period to assess food intake by a 24-hour dietary recall, as described elsewhere (73). Nutrient intake from these dietary records and recalls was determined using a nutrient software program (Compl-eat, Human Nutrition Wageningen University, Wageningen, Netherlands). Third, free AGEs in 24-hour urine samples and plasma were compared between groups after the intervention.

*Experimental visits*. All measurements were performed by the head investigator. Measurements were conducted in a temperature-controlled room (24°C ± 0.5°C) after a 12-hour overnight fast and a 30-minute acclimatization period. Participants were instructed to refrain from alcohol and strenuous physical exercise for a period of 48 hours prior to each study day. Prior to the microvascular measurements, 2 venous catheters were fitted: one for sample collection, and the other for delivery of venous infusion. The infusion cannula was fitted in an antecubital vein of the left arm. The sampling cannula was placed in a suitable wrist vein of the ipsilateral hand, if possible, or an antecubital vein of the right arm.

*Hyperinsulinemic euglycemic clamp*. Insulin sensitivity was assessed by a 1 mU/kg/min euglycemic insulin clamp as described previously (74). Briefly, insulin (NovoRapid, Novo Nordisk) was infused in a primed continuous manner for 120 minutes. Meanwhile, isoglycemia was maintained with a variable rate 20% glucose infusion. Metabolic insulin sensitivity was estimated from the steady-state glucose infusion rate (90–120 minutes of the clamp). Plasma glucose concentrations were measured in centrifuged venous blood samples (13,000*g* for 30 seconds at room temperature) with an on-site YSI2300 glucose analyzer (YSI).

Insulin clearance was determined in the fasting state and during the steady-state period from the hyperinsulinemic clamp. Fasting insulin clearance was calculated as the ratio between fasting insulin secretion, determined by C-peptide deconvolution (43), and fasting plasma insulin concentration. Insulin clearance during the steady-state period of the hyperinsulinemic clamp was calculated as the ratio between insulin infusion rate and plasma insulin concentrations during the 90- to 120-minute period. Plasma insulin concentrations were adjusted for endogenously secreted insulin, which was determined with the following formula:

Endogenously-secreted insulin *=* insulin_fasting_ × (C-peptide_90-120_/C-peptide_fasting_).

This formula assumes that the endogenously secreted insulin during the 90- to 120-minute period of the hyperinsulinemic clamp changes in proportion to the C-peptide change in this period.

### C-peptide suppression, reflecting insulin’s potential to inhibit its own secretion (39), was calculated as percentage change in average C-peptide concentration during the 90- to 120-minute period relative to fasting values, using the following formula:

C-peptide suppressio*n =* ([C-peptide_90-120_ – C-peptide_fasting_]/C-peptide_fasting_) × 100%.

#### Hyperglycemic clamp.

β Cell function was determined by a hyperglycemic clamp as described previously (75). After the hyperinsulinemic euglycemic clamp, insulin infusion was discontinued, and the glucose infusion rate was gradually decreased over a period of 60 minutes while fasting glucose levels were maintained. Next, a 30-minute square-wave step of hyperglycemia was applied to assess β cell function. This was achieved by a priming glucose dose (2.8 mmol/L above baseline), administered over 1 minute, followed by a variable 20% glucose infusion to maintain plasma glucose concentrations at the desired plateau. Venous blood samples for determination of plasma glucose, insulin, and C-peptide were obtained every 2 minutes for the first 10 minutes and every 5 minutes for the remainder of the step.

ISRs were calculated by means of C-peptide deconvolution (43). The first-phase ISR response to the i.v. glucose bolus was expressed as the mean ISR incremental AUC during the first 8 minutes after the glucose bolus. The second-phase ISR response to the hyperglycemic clamp was expressed as the mean ISR AUC during 10–30 minutes. Because plasma glucose reached steady state mostly during the end of the hyperglycemic clamp, the second-phase ISR response was also expressed as the mean ISR AUC during the last 5 minutes. Finally, β cell glucose sensitivity was expressed as the ratio between the ISR increment from baseline to 25–30 minutes and the corresponding glucose increment.

#### Contrast enhanced ultrasound.

IMMR in forearm skeletal muscle during acute hyperinsulinemia was measured as described previously (74). In short, microvascular blood volume of forearm skeletal muscle was measured with a Toshiba Aplio XG ultrasound system (Toshiba) during continuous infusion of sulfur hexafluoride gas-filled microbubbles (SonoVue, Bracco Diagnostics) in the fasting state and after 100 minutes of hyperinsulinemia. After 3 minutes of microbubble infusion, a steady state microbubble concentration was achieved, and 5 real-team replenishment curves of 30 seconds were obtained after microbubble disruption by a high mechanical index ultrasound pulse. These replenishment curves were stored and analyzed offline in a blinded fashion using the CHI-Q software (Toshiba). The replenishment curves were fitted to the exponential function γ = A(1 – e^–βt^) — where t is the pulsing interval, γ is the video intensity at any given t, A is the plateau video intensity, and β is microvascular flow velocity — and averaged as described elsewhere (76). IMMR was expressed as the relative increase in microvascular blood volume during hyperinsulinemia. Collection of other microvascular measurements, and macrovascular measurements, is explained in detail in the Supplemental Methods.

#### Measurements in plasma.

Soluble vascular cell adhesion molecule-1 (sVCAM-1), sICAM-1, high sensitivity C-reactive protein (CRP), tumor necrosis factor α (TNF-α), IL-6, IL-8, adiponectin, serum amyloid A (SAA), insulin, and C-peptide were measured in EDTA plasma samples with commercially available 4-plex sandwich immunoassay kits (Meso Scale Discovery [MSD]), as described elsewhere (77). Soluble E-selectin (sE-selectin) was measured in EDTA plasma samples with sandwich ELISA (Diaclone). von Willebrand factor (vWf) was determined in citrated plasma with sandwich ELISA (Dako). Concentrations of vWf were expressed as a percentage of vWf detected in pooled citrated plasma of healthy volunteers. γ-GT, total cholesterol, HDL cholesterol, and triglycerides were determined in serum using enzymatic and colorimetric methods by an automatic analyzer (Beckman Synchron LX20, Backman Coulter Inc.). LDL cholesterol was determined via the Friedewald formula (78).

#### AGEs, dicarbonyls, and oxidative stress markers in plasma and urine.

Free and protein-bound AGEs in plasma and free AGEs in urine were analyzed as described in detail elsewhere (79). In brief, for protein-bound and free AGEs in plasma, 25 μL and 50 μL of plasma was used, respectively. For free AGEs in urine, 40 μL of urine were used. All samples were derivatized with butanolic hydrochloric acid and subsequently detected in electrospray ionization–positive (ESI^+^) multiple-reaction monitoring (MRM) mode using a Xevo TQ MS (Waters). Quantification of CML, CEL, and MG-H1 was performed by calculating the peak area ratio of each unlabeled peak area to the corresponding internal standard peak area. In plasma, the intra- and interassay variation of protein-bound CML and CEL was between 4.8% and 9.7% and — for free CML, CEL, and MG-H1 — between 2.8% and 7.1%. In urine, the intra- and interassay variation of free CML, CEL, and MG-H1 was between 3.7% and 6.6%. Measurement of dicarbonyls in plasma was performed as described in detail elsewhere (80) Measurement of AGE-accumulation in skin-by-skin autofluorescence is described in detail in the Supplemental Methods.

For the quantification of the urinary biomarkers CML, CEL, MG-H1, pyrraline, MGO, 8-Oxo-dG, and CEdG, 50 μL of urine was mixed with internal standard mix and derivatized with acidified o-Phenylenediamine and subsequently separated on a UPLC C18-column using ion-pair solvents. All biomarkers were detected in positive MRM, with MGO as a quinoxaline adduct. All biomarkers were successfully separated and detected with UPLC-MS/MS with a run-to-run time of 14 minutes. Linearity of all markers was tested in a water and urine matrix and showed good correlation (*r*^2^ > 0.99) with an intra- and interassay coefficient of variations (CV) of about 5%.

#### Statistics.

Analyses were conducted using a prespecified analysis plan, blinded for randomization, using SPSS version 25 for Windows (IBM Corp.). Data are presented as means ± SD, medians (IQR), or percentages, as appropriate. Outcomes were assessed in an intention-to-treat complete case analysis. Differences within groups after the intervention were assessed by a paired-samples 2-tailed *t* test, whereas differences between groups after the intervention period were assessed by a 1-way ANCOVA with sex, age, and the baseline value of the outcome of interest as a covariate. *P* < 0.05 was considered statistically significant.

We investigated the robustness of our findings in sensitivity analyses. To adjust for differences in macronutrient content between intervention diets, we additionally adjusted the ANCOVA model for intake of carbohydrate, protein, and fat as energy percentages. Secondly, we used multiple linear regression analysis to investigate whether indices of dietary AGE intake, the state of being self-assessed AGE intake during the intervention, and free AGEs in plasma and urine were also associated with outcomes. The fully adjusted regression model was adjusted for age, sex, and intake of carbohydrate, protein, and fat as energy percentages.

#### Study approval.

This study was approved by the Maastricht University Medical Center ethics committee, performed in accordance with the Declaration of Helsinki, and registered at both international and national trial registries (clinicaltrials.gov, NCT03866343; trialregister.nl, NTR7594). All participants provided written informed consent.

## Author contributions

SJMPE, AJHMH, CDAS, and CGS designed the study; AMAL, PMN, NEGW, EECDG, and MDGVDE conducted research; MCJMVD and CCJAWVG performed randomization; AMAL, AJHMH, SJPME, NEGW, AM, JLJMS, MPHVDW, LS, HH, and TTJMB analyzed data; AMAL performed statistical analyses and wrote the paper; and CGS had the primary responsibility for the final content.

## Supplementary Material

Supplemental data

## Figures and Tables

**Table 1:**
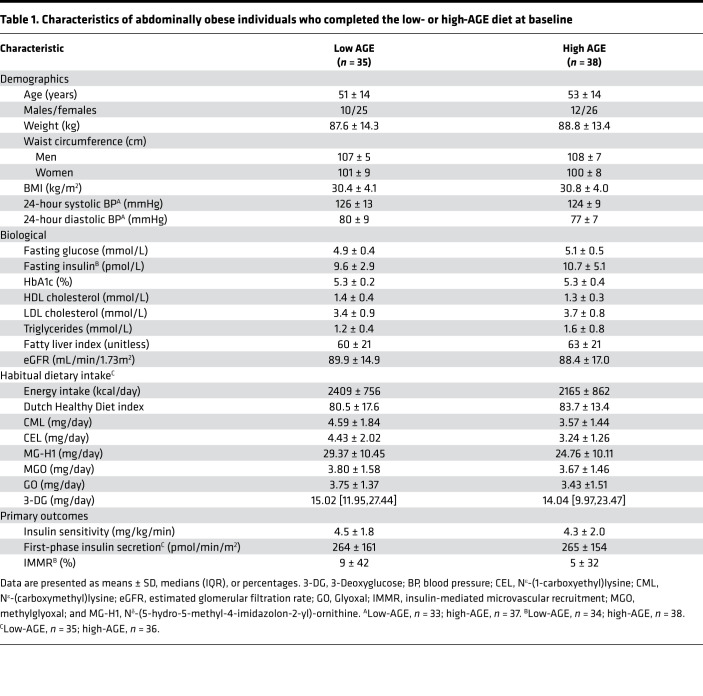
Characteristics of abdominally obese individuals who completed the low- or high-AGE diet at baseline

**Table 2:**
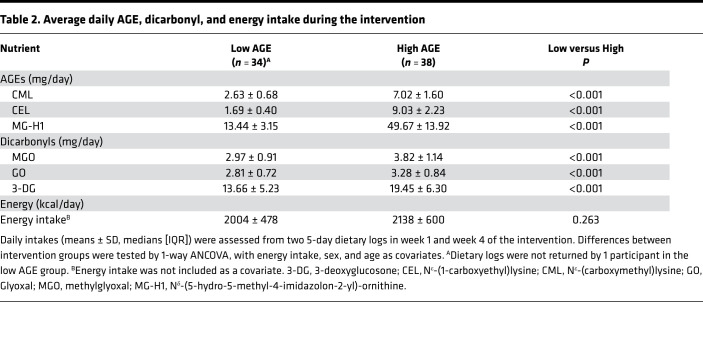
Average daily AGE, dicarbonyl, and energy intake during the intervention

**Table 3:**
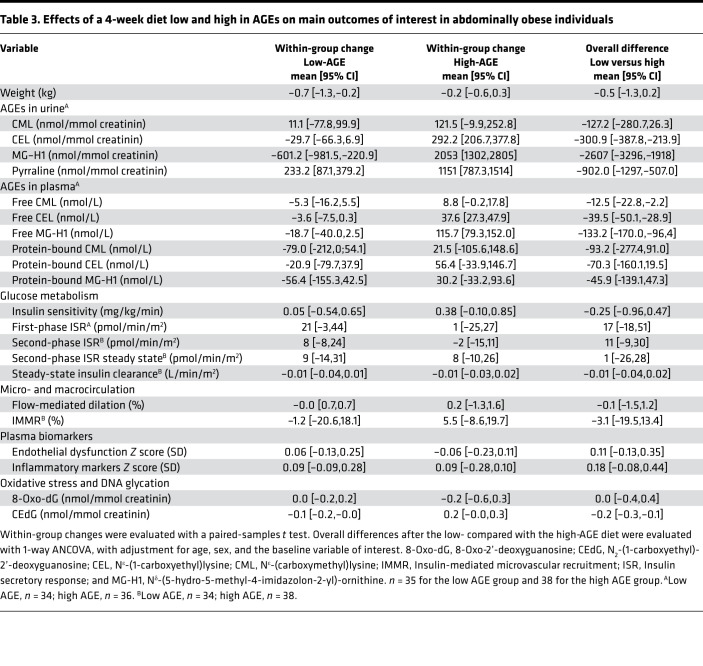
Effects of a 4-week diet low and high in AGEs on main outcomes of interest in abdominally obese individuals

## Version 1 02/08/2022

In-Press Preview

## Version 2 03/22/2022

Electronic publication
