# Supplementary material for: A 4-week high-AGE diet does not impair glucose metabolism and vascular function in obese individuals
Source: JCI Insight. 2022 Mar 22;7(6):e156950. doi: 10.1172/jci.insight.156950 (PMC8986074; doi:10.1172/jci.insight.156950)
Supplement: Supplemental data [file jciinsight-7-156950-s265.pdf]

Supplemental Methods

Macrocirculation

A 3-lead electrocardiogram (ECG) was recorded continuously during the measurements to facilitate automatic signal processing. During arterial stiffness measurement, repeated brachial systolic, diastolic, and mean arterial pressures were obtained at 5-minute intervals, using an oscillometric device (Accutorr Plus, Datascope, Inc, Montvale, NJ). The time-averages of systolic, diastolic, and mean arterial pressure were used in the analysis.

Carotid-to-femoral Pulse Wave Velocity

Aortic stiffness was determined by measuring carotid-to-femoral pulse wave velocity (cfPWV) according to recent guidelines (1) with the use of applanation tonometry (SphygmoCor, Atcor Medical, Sydney, Australia). Pressure waveforms were determined at the left common carotid and left common femoral arteries. The difference in the time of pulse arrival from the R-wave of the ECG between the 2 sites (transit time) was determined with the intersecting tangents algorithm. The pulse wave travel distance was calculated as 80% of the direct straight distance (measured with an infantometer) between the 2 arterial sites. cfPWV was defined as travelled distance/transit time. We used the median of 3 consecutive cfPWV recordings in the analyses. Where more than 3 measurements were performed, the measurements with the lowest standard deviation were used.

Carotid Distensibility coefficient and Young’s elastic modulus

Indices of carotid stiffness were measured at the left common carotid artery (10 mm proximal to the carotid bulb), with the use of an ultrasound scanner equipped with a

7.5-MHz linear probe (MyLab 70, Esaote Europe B.V., Maastricht, the Netherlands). This set-up enables the measurement of diameter, distension, and intima-media thickness (IMT) as described previously (2,3). Briefly, during the ultrasound measurements, a B-mode image based on 19 M-lines was displayed on screen. An online echo-tracking algorithm showed real-time anterior and posterior wall displacements. The multiple M-line recordings were composed of 19 simultaneous recordings at a frame rate of 498 Hz. The distance between the M-line recording positions was 0.96 mm; thus, a total segment of 18.24 mm of each artery was covered by the scan plane. For offline processing, the radiofrequency signal was acquired by a dedicated computer-based system (ART.LAB, Esaote Europe B.V. Maastricht, the Netherlands) with a sampling frequency of 50 MHz. Data processing was performed in MatLab (version 7.5; Mathworks, Natick, Massachusetts, USA). Distension waveforms were obtained from the radiofrequency data by wall tracking, as described in (2). We defined carotid IMT as the distance of the posterior wall from the leading edge interface between lumen and intima to the leading edge interface between media and adventitia (3). We used the median diameter, median distension and median IMT of three recordings in the analyses.

Data analysis was done by quantifying the local arterial elastic properties through the calculation of the following indices (4):

1. Distensibility coefficient (carDC) = $(2\Delta D \times D + \Delta D^2)/(\mathrm{PP} \times D^2)$ (10^{-3}/kPa)
2. Young's elastic modulus (carYEM) = $D/(\mathrm{IMT} \times \text{distensibility coefficient})$ (10^3 kPa)

where *D* is the arterial diameter; Δ*D* is the distension; IMT the intima–media thickness; and PP the pulse pressure. Local carotid PP was estimated according to the calibration method described by Kelly and Fitchett (5), with the use of carotid tonometry waveforms as adapted by van Bortel *et al.* (6). This method assumes a constant difference between MAP and diastolic pressure along the arterial tree. PP can then be calculated at a carotid artery (PPcar) from the uncalibrated carotid pressure waveform using the formula: $PPcar = PPcar_{uncalibrated} \times (Kbrach/Kcar_{uncalibrated})$, in which K is defined as (MAP − diastolic pressure). For the carotid artery, diastolic pressure and MAP are calculated as the minimum and the area under the tonometry waveform divided by time, respectively. The carDC reflects the inverse of arterial stiffness at operating pressure. The carYEM reflects the stiffness of the arterial wall material at operating pressure.

Note that increased values of cfPWV or of carYEM, or decreased values of carDC, indicate increased central arterial stiffness.

Radial Pulse Wave Analysis

Radial artery pulse wave analysis was measured in triplicate at the wrist of the right arm using tonometry (SphygmoCor v9; AtCor Medical), as described previously (7). In short, the central arterial waveform was derived from the peripheral arterial waveform using a validated transfer function. The augmentation index was defined as the difference between the first and second peak of the central arterial waveform, expressed as a percentage of the pulse pressure and corrected for heart rate. We used the median of 3 consecutive measurements. As in some participants more than 3 measurements were performed, we chose the measurements with the highest quality based on four criteria (8): (1) average pulse height above 100 units, (2) pulse height

variation < 5%, (3) diastolic variation < 5%, and (4) systolic peak between 80 and 150 ms from the start of the wave. Measurements were scored on a scale from 0 to 4 based on the number of criteria met. Measurements with low quality scores (0 or 1) were excluded from the analysis. For one participant, this resulted in 2 measurements instead of 3, and these 2 measurements were averaged instead of using the median.

Flow-mediated dilation

Flow-mediated dilation (FMD) of the brachial artery was assessed by ultrasound echography in dual mode (MyLab70, Esaote) and recording of echo images on DVD, as described previously (7). These images were analyzed offline using a custom-written Matlab program (MyFMD; AP Hoeks, Department of Biomedical Engineering, Maastricht University Medical Center, Maastricht, the Netherlands). After a 5-minute reference period, a pneumatic cuff placed around the participant's right forearm was inflated to 200 mmHg for 5 minutes to ensure arterial occlusion. After 5 minutes of arterial occlusion, the cuff was deflated and images were obtained for an additional 5 minutes. The FMD response was quantified as the maximal percentage change in post occlusion arterial diameter relative to the baseline diameter.

Microcirculation

Laser Doppler Flowmetry

Skin blood flow was measured both in the basal state, during acute hyperinsulinemia, and during acute local heating, as described previously, by means of a laser-Doppler system (Periflux 5000; Perimed, Järfalla, Sweden) equipped with two thermostatic laser-Doppler probes (PF457; Perimed) at the dorsal side of the wrist of the left hand (9). The laser-Doppler output was recorded for 35 minutes with a sample rate of 32 Hz,

which gives semi quantitative assessment of skin blood flow expressed in arbitrary perfusion units.

Flowmotion

Since skin microvascular flowmotion (SMF) has predominantly been observed in participants with a skin temperature above 29.3°C (10), the laser-Doppler probe was set at 30°C. The skin blood flow signal was transformed into five different SMF components by means of a Fast-Fourier transform algorithm using dedicated custom build software (FlowPSD; AP Hoeks, Department of Biomedical Engineering, Maastricht University Medical Center, Maastricht, the Netherlands). The frequency spectrum between 0.01 and 1.6 Hz was divided into five components: (1) endothelial, 0.01-0.02 Hz, (2) neurogenic, 0.02-0.06 Hz, (3) myogenic, 0.06-0.15 Hz, (4) respiratory, 0.15-0.40 Hz, and (5) heartbeat, 0.40-1.60 Hz (11). Additionally, total SMF energy was obtained by the sum of the power density values of the total frequency spectrum.

Heat-induced skin hyperemic response

With the second probe, skin blood flow was first recorded unheated for 2 minutes to serve as a baseline. After the 2 minutes of baseline, the temperature of the probe was rapidly and locally increased to 44°C and was then kept constant until the end of the registration. The heat-induced skin hyperemic response was expressed as the percentage increase in average perfusion units during the 33-minute heating phase over the average baseline perfusion units.

Retinal imaging

Fundus photographs were obtained to assess static retinal microvascular diameters with a non-mydriatic manual-focus fundus camera (Canon). To this end, three optic-disc centered photographs of the right eye were taken. The detailed procedure has been explained elsewhere (12). In short, retinal arteriolar and venular diameters were measured at an area 0.5-1.0 disc diameter away from the optic disc margin with semi-automatic analyzing software (Vesselmap 3.0, Visualis, Imedos Systems UG). Arteriolar and venular diameters were averaged to central retinal arteriolar (CRAE) and venular (CRVE) equivalents using the Parr-Hubbard formula (13). Vessel diameters are presented in µm, as one measuring unit of the imaging device relates to 1 µm in the model of Gullstrand‘s normal eye. The same researcher took all images, and all images were analysed by the same independent researcher, unaware of a participant’s treatment allocation. Participants with retinal pathologies that influence microvascular calibers (e.g. macular degeneration, $n = 1$) were excluded from the analyses.

Habitual food intake and dietary advanced glycation endproducts

We assessed habitual dietary intake by a validated 253-item food frequency questionnaire (FFQ) (14). This FFQ contains 101 questions on consumption with a reference period of one year. The FFQ collected information on the intake of major food groups. Food intake was determined by the combination of frequency questions with quantity questions. For the frequency questions, 11 options were available ranging from “not used” to 7 days/week. For the quantity questions, variable options were available based on fourteen standard household servings, ranging from < 1/day to > 12/day. Average daily consumption of food items was then calculated by multiplying the frequency and amount. Energy and nutrient intakes were subsequently determined

by transcribing food items into food codes embedded in the Dutch Food Composition Table 2011 (15). Additionally, we determined the Dutch Healthy Diet (DHD) index based on this food intake data. The DHD-index is a measure of diet quality as it assesses adherence to the Dutch dietary guidelines (16). A higher index has been associated with more nutrient-dense diets and lower risk of mortality (17,18).

Dietary AGE intake was determined by coupling the consumption of food items within the FFQ to our dietary AGE database (19). In this database, three major AGEs, CML, CEL, and MG-H1, were quantified in protein fractions of food products using highly specific ultra performance liquid chromatography tandem mass spectrometry (UPLC-MS/MS). In total, this database includes over 200 food products commonly consumed in a Western diet. For each participant, AGE intake was estimated as described previously (20). Some of the food products in the FFQ were not analyzed for AGEs content. AGE content of these specific products were estimated by matching them to other products that were comparable in macronutrient profile and preparation method. For example, for several fresh vegetables boiled in water, such as endive, beets, leek, and spinach, the same AGE content was used. By comparison, jarred peas and carrots were measured separately from fresh peas and carrots, as AGEs in jarred peas and carrots are higher as they contain added sugar and are heated to prolong shelf life (19).

Skin autofluoresence

Skin autofluoresence (SAF) was measured with the AGE Reader (DiagnOptics Technologies BV, Groningen, The Netherlands). The AGE reader is a desktop device that uses the characteristic fluorescent properties of certain AGEs to estimate the level of AGE accumulation in the skin. Technical details of this noninvasive method have

been described more extensively elsewhere (21). In short, the AGE Reader illuminates a skin surface of 4 cm2 guarded against surrounding light, with an excitation wavelength range of 300 to 420 nm, with a peak excitation of 370 nm. SAF was calculated as the ratio between the emission light from the skin in the wavelength range of 420 to 600 nm (fluorescence) and excitation light that is reflected by the skin (300–420 nm), multiplied by 100 and expressed in arbitrary units. Participants were asked not to use any sunscreen or self-browning creams on their lower arms within 2 days before the measurement. SAF was measured at room temperature in a semidark environment, where participants were at rest in a seated position. The inner side of the forearm ≈4 cm below the elbow fold of a participant was positioned on top of the device, as described by the manufacturer. The mean of 3 consecutive measurements was used in the analyses.

References to supplemental material

1. Van Bortel LM, Laurent S, Boutouyrie P, et al. Expert consensus document on the measurement of aortic stiffness in daily practice using carotid-femoral pulse wave velocity. *Journal of hypertension*. Mar 2012;30(3):445-8. doi:10.1097/HJH.0b013e32834fa8b0
2. Hermeling E, Reesink KD, Kornmann LM, Reneman RS, Hoeks AP. The dicrotic notch as alternative time-reference point to measure local pulse wave velocity in the carotid artery by means of ultrasonography. *Journal of hypertension*. Oct 2009;27(10):2028-35. doi:10.1097/HJH.0b013e32832f5890
3. Willekes C, Hoeks AP, Bots ML, Brands PJ, Willigers JM, Reneman RS. Evaluation of off-line automated intima-media thickness detection of the common carotid artery based on M-line signal processing. *Ultrasound in medicine & biology*. Jan 1999;25(1):57-64.
4. Reneman RS, Meinders JM, Hoeks AP. Non-invasive ultrasound in arterial wall dynamics in humans: what have we learned and what remains to be solved. *European heart journal*. May 2005;26(10):960-6. doi:10.1093/eurheartj/ehi177
5. Kelly R, Fitchett D. Noninvasive determination of aortic input impedance and external left ventricular power output: a validation and repeatability study of a new technique. *Journal of the American College of Cardiology*. Oct 1992;20(4):952-63. doi:10.1016/0735-1097(92)90198-v
6. Van Bortel LM, Balkestein EJ, van der Heijden-Spek JJ, et al. Non-invasive assessment of local arterial pulse pressure: comparison of applanation tonometry and echo-tracking. *Journal of hypertension*. Jun 2001;19(6):1037-44.
7. Joris PJ, Plat J, Kusters YH, et al. Diet-induced weight loss improves not only cardiometabolic risk markers but also markers of vascular function: a randomized controlled trial in abdominally obese men. *The American journal of clinical nutrition*. Jan 2017;105(1):23-31. doi:10.3945/ajcn.116.143552
8. Janner JH, Godtfredsen NS, Ladelund S, Vestbo J, Prescott E. Aortic augmentation index: reference values in a large unselected population by means of the SphygmoCor device. *American journal of hypertension*. Feb 2010;23(2):180-5. doi:10.1038/ajh.2009.234
9. Muris DM, Houben AJ, Kroon AA, et al. Age, waist circumference, and blood pressure are associated with skin microvascular flow motion: the Maastricht Study. *Journal of hypertension*. Dec 2014;32(12):2439-49; discussion 2449. doi:10.1097/HJH.0000000000000348
10. Thorn CE, Kyte H, Slaff DW, Shore AC. An association between vasomotion and oxygen extraction. *American journal of physiology Heart and circulatory physiology*. Aug 2011;301(2):H442-9. doi:10.1152/ajpheart.01316.2010
11. Stefanovska A, Bracic M, Kvernmo HD. Wavelet analysis of oscillations in the peripheral blood circulation measured by laser Doppler technique. *IEEE transactions on bio-medical engineering*. Oct 1999;46(10):1230-9.
12. Streese L, Deiseroth A, Schafer J, Schmidt-Trucksass A, Hanssen H. Exercise, Arterial Crosstalk-Modulation, and Inflammation in an Aging Population: The ExAMIN AGE Study. *Front Physiol*. 2018;9:116. doi:10.3389/fphys.2018.00116
13. Hubbard LD, Brothers RJ, King WN, et al. Methods for evaluation of retinal microvascular abnormalities associated with hypertension/sclerosis in the Atherosclerosis Risk in Communities Study. *Ophthalmology*. Dec 1999;106(12):2269-80. doi:10.1016/s0161-6420(99)90525-0
14. van Dongen MC, Wijckmans-Duysens NEG, den Biggelaar LJ, et al. The Maastricht FFQ: Development and validation of a comprehensive food frequency questionnaire for the Maastricht study. *Nutrition*. Jun 2019;62:39-46. doi:10.1016/j.nut.2018.10.015
15. RIVM/Voedingscentrum. Dutch Food Composition Table (NEVO). 2011. Accessed February 6th, 2019. https://www.rivm.nl/neder-lands-voedingsstoffenbestand/toegang-nevo-gegevens/nevo-online/zoeken-bekijken-en-vergelijken

16. Looman M, Feskens EJ, de Rijk M, et al. Development and evaluation of the Dutch Healthy Diet index 2015. *Public Health Nutr*. Sep 2017;20(13):2289-2299. doi:10.1017/S136898001700091X
17. van Lee L, Feskens EJ, Hooft van Huysduynen EJ, de Vries JH, van 't Veer P, Geelen A. The Dutch Healthy Diet index as assessed by 24 h recalls and FFQ: associations with biomarkers from a cross-sectional study. *J Nutr Sci*. 2013;2:e40. doi:10.1017/jns.2013.28
18. van Lee L, Geelen A, Kiefte-de Jong JC, et al. Adherence to the Dutch dietary guidelines is inversely associated with 20-year mortality in a large prospective cohort study. *European journal of clinical nutrition*. Feb 2016;70(2):262-8. doi:10.1038/ejcn.2015.163
19. Scheijen J, Clevers E, Engelen L, et al. Analysis of advanced glycation endproducts in selected food items by ultra-performance liquid chromatography tandem mass spectrometry: Presentation of a dietary AGE database. *Food chemistry*. Jan 1 2016;190:1145-1150. doi:10.1016/j.foodchem.2015.06.049
20. Scheijen J, Hanssen NMJ, van Greevenbroek MM, et al. Dietary intake of advanced glycation endproducts is associated with higher levels of advanced glycation endproducts in plasma and urine: The CODAM study. *Clinical nutrition*. Jun 2018;37(3):919-925. doi:10.1016/j.clnu.2017.03.019
21. Meerwaldt R, Graaff R, Oomen PHN, et al. Simple non-invasive assessment of advanced glycation endproduct accumulation. *Diabetologia*. Jul 2004;47(7):1324-1330. doi:10.1007/s00125-004-1451-2

Supplemental Figures

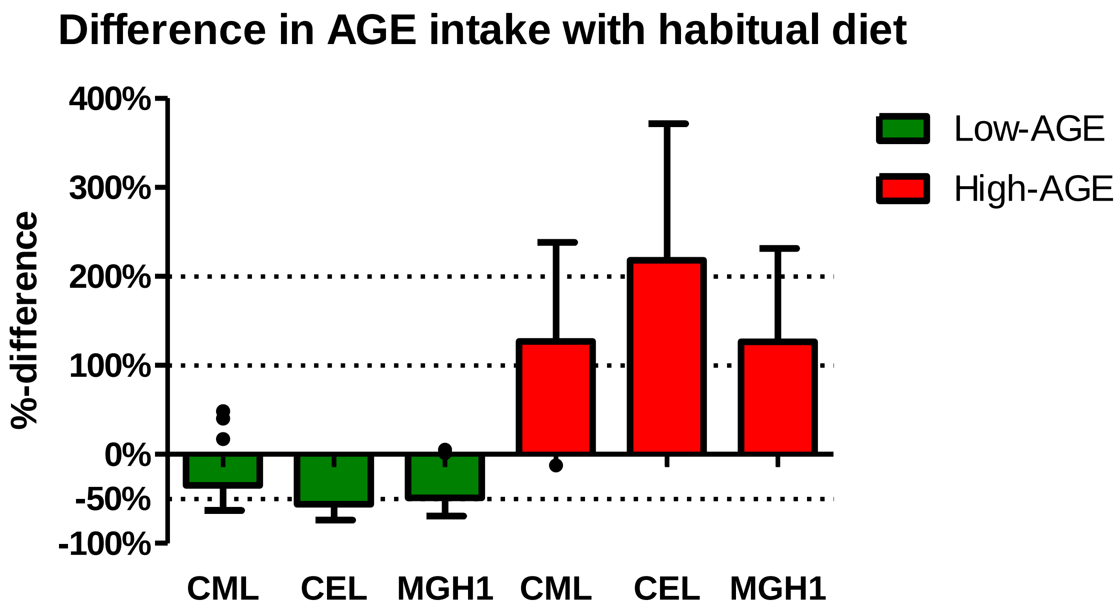

Supplemental Figure 1 Percentage difference in intake of AGEs between the habitual diet (assessed with food frequency questionnaires) and during the intervention (assessed as the average daily intake from two five-day dietary logs). Bar plots indicate mean ± SD. Black circles indicate individuals that showed no increase or decrease in respective AGE intake during their intervention. *n*=34 for the low-AGE group, *n*=38 for the high-AGE group.

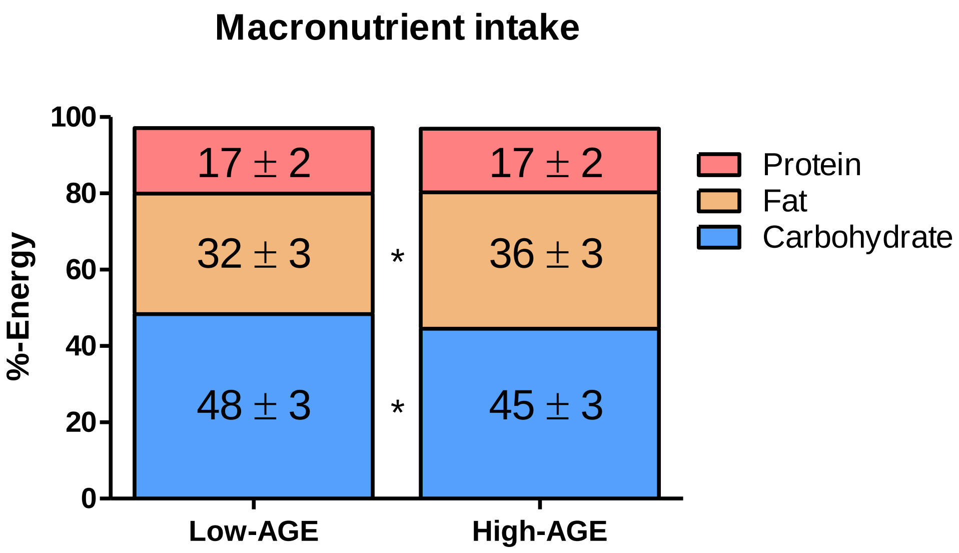

Supplemental Figure 2 Difference in macronutrient intake (as percentage-energy) during the low- and high-AGE diet. * indicates a difference between intervention diets at the < 0.05 level. Fiber intake represented 2%, while alcohol intake represented 1% of percentage-energy in both groups.

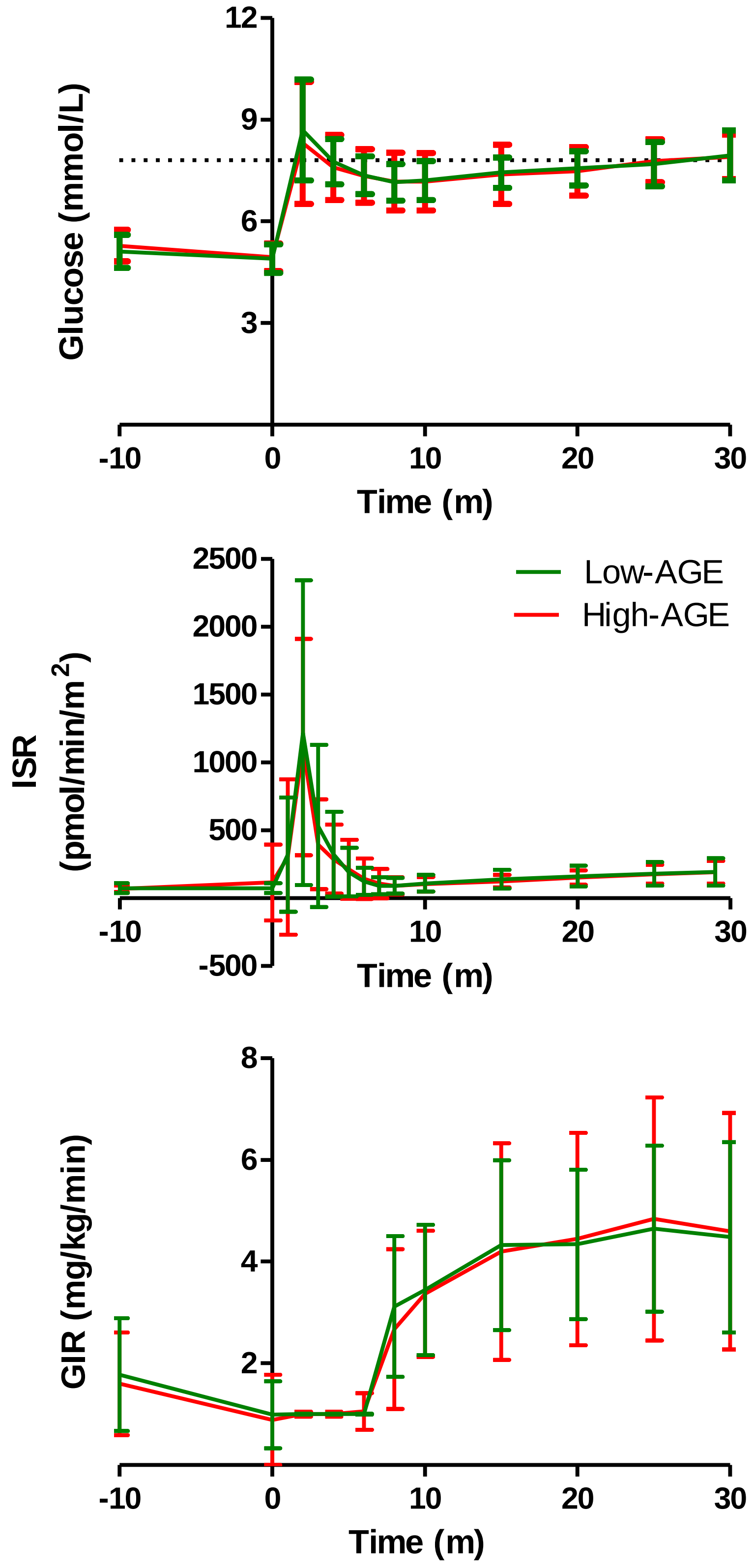

Supplemental Figure 3 Hyperglycemic clamp results at follow-up (*n*=72). Upper: plasma glucose after administration of the glucose bolus and subsequent clamping at 2.8 mmol/L above fasting values (dotted line). Middle: ISR profile. Lower: Glucose infusion rate. Abbrevations: GIR: glucose infusion rate. ISR: insulin secretion rate.

Supplemental Tables

Supplemental Table 1 Comparison of baseline characteristics from participants included in the complete case analysis to those excluded resulting from missing the primary outcome.

Characteristic	Primary outcome at follow-up not collected (n=9)	Primary outcome at follow-up collected (*n*=73)
**Demographics**[1]		
Age (years)	46 ± 19	52 ± 14
Males/Females	1/8	22/51
Weight (kg)	89.1 ± 3.6	88.3 ± 13.7
Waist circumference (cm)		
Men	108.0 ± 0.0	107.3 ± 5.9
Women	99.5 ± 6.7	100.6 ± 8.2
BMI (kg/m^2)	29.8 ± 2.8	30.6 ± 4.0
24-hour systolic BP[2] (mmHg)	112 ± 12	125 ± 11
24-hour diastolic BP[2] (mmHg)	75 ± 6	78 ± 8
**Biological**		
Fasting glucose (mmol/L)	5.3 ± 0.36	5.0 ± 0.5
Fasting insulin (pmol/L)	8.3 ± 0.9	10.1 ± 4.2
HbA1c[1] (%)	5.3 ± 0.4	5.3 ± 0.3
HDL cholesterol (mmol/L)	1.4 ± 0.25	1.4 ± 0.4
LDL cholesterol (mmol/L)	3.4 ± 1.1	3.5 ± 0.8
Triglycerides (mmol/L)	1.4 ± 0.8	1.4 ± 0.7
Fatty liver index (unitless)	57 ± 25	61 ± 22
eGFR (ml/min/1.73m^2)	86.4 ± 21.5	89.1 ± 16.0
**Habitual dietary intake**[3]		
Energy intake (kcal/day)	2116 ± 476	2286 ± 814
Dutch Healthy Diet index	73.7 ± 12.7	82.1 ± 15.6
CML (mg/day)	3.23 ± 0.33	4.07 ± 1.71
CEL (mg/day)	3.00 ± 0.28	3.83 ± 1.77
MG-H1 (mg/day)	21.34 ± 4.56	27.03 ± 10.46
MGO (mg/day)	3.07 ± 0.43	3.73 ± 1.51
GO (mg/day)	2.93 ± 0.53	3.59 ± 1.441
3-DG (mg/day)	9.97 [7.41,-]	15.00 [10.84,25.94]
**Primary outcomes**		
Insulin sensitivity[1] (mg/kg/min)	6.7 ± 4.4	4.4 ± 1.9
First-phase insulin secretion[3] (pmol/min/m^2)	161 ± 88	263 ± 155
IMMR[4] (%)	-	7 ± 36

Data are presented as means ± SD, medians [interquartile range], or percentages. Abbreviations: 3-DG: 3-Deoxyglucose. BP: blood pressure. CEL: N^{ε}-(1-carboxyethyl)lysine. CML: N^{ε}-(carboxymethyl)lysine. eGFR: estimated glomerular filtration rate. GO: Glyoxal. IMMR: Insulin-mediated microvascular recruitment. MGO: Methylglyoxal. MG-H1: N^{δ}-(5-hydro-5-methyl-4-imidazolon-2-yl)-ornithine.

[1] n=7 for those missing, *n*=73 for those included.

[2] n=7 for those missing, *n*=71 for those included.

[3] n=3 for those missing, *n*=72 for those included.

[4] *n*=0 for those missing, *n*=72 for those included.

Supplemental Table 2 Average daily AGE-, dicarbonyl- and energy- intake during the intervention as determined by two 24-hour recalls

Nutrient	Low AGE (n=37)[1]	High AGE (n=37)	Low vs High ***p***
**AGEs (mg/day)**			
CML	2.67 ± 0.96	6.66 ± 3.40	<0.001
CEL	1.65 ± 0.68	8.31 ± 7.20	<0.001
MG-H1	13.72 ± 3.78	47.70 ± 20.84	<0.001
**Dicarbonyls (mg/day)**			
MGO	2.85 ± 0.93	3.66 ± 1.12	0.001
GO	2.87 ± 0.90	3.27 ± 0.77	0.027
3-DG	14.22 ± 7.04	17.42 ± 9.42	0.231
**Energy (kcal/day)**			
Energy intake[1]	2050 ± 500	2162 ± 621	0.367

Daily intakes (means ± SD, medians [IQR]) were assessed from two 24-hour recalls in week 3 and week 4 of the intervention. Differences between intervention groups were tested by a one-factor ANCOVA with energy intake, sex, and age as covariates.
[1]Energy intake was not included as a covariate.

Supplemental Table 3 Average daily micronutrient intake during the low and high AGE diets

Micronutrient	Low AGE (n=34)[1]	High AGE (n=38)	Low vs High ***p***
**Vitamins**			
Retinol activity equivalents (µg/day)	1579.4 ± 547.2	534.7 ± 172.4	<0.001
Thiamine (mg/day)	1.1 ± 0.2	1.1 ± 0.2	0.039
Nicotinic acid (mg/day)	17.8 ± 3.8	18.2 ± 4.7	0.179
Pyridoxin (mg/day)	1.5 ± 0.3	1.3 ± 0.3	<0.001
Folate equivalents (µg/day)	225.5 ± 34.1	224.1 ± 51.1	0.026
Cobalamin (µg/day)	4.8 ± 0.9	4.4 ± 1.0	<0.001
Ascorbic acid (µg/day)	67.9 ± 16.3	73.9 ± 16.4	0.298
Cholecalciferol (µg/day)	4.1 ± 1.3	5.4 ± 2.1	0.005
Calcidiol (µg/day)	0.3 ± 0.2	0.3 ± 0.1	0.758
Tocopherols and tocotrienols (mg/day)	9.1 ± 1.8	12.3 ± 1.8	<0.001
**Minerals**			
Calcium (mg/day)	1031.9 ± 172.8	1002.3 ± 230.9	0.014
Phosphor (mg/day)	1496.5 ± 257.1	1616.2 ± 380.2	0.097
Total iron (mg/day)	11.3 ± 3.1	11.5 ± 3.0	0.208
Sodium (mg/day)	2286.2 ± 464.3	2647.4 ± 741.2	<0.001
Potassium (mg/day)	3252.8 ± 652.1	3444.7 ± 721.6	0.781
Magnesium (mg/day)	331.6 ± 70.0	385.1 ± 96.1	<0.001
Zink (mg/day)	10.7 ± 2.0	10.4 ± 2.5	<0.001
Selenium (µg/day)	47.7 ± 8.4	53.5 ± 15.3	0.041
Copper (mg/day)	1.2 ± 0.3	1.5 ± 0.4	<0.001
Iodine (µg/day)	211.7 ± 43.2	200.5 ± 53.1	<0.001

Daily intakes (means ± SD, medians [IQR]) of micronutrients were assessed from two five-day dietary logs at week 1 and week 4 of the intervention. Differences between intervention groups were tested by a one-factor ANCOVA with energy intake, sex, and age as covariates.
[1]Dietary logs were not returned by one participant in the low AGE group.

Supplemental Table 4 Effects of a 4-week low- and high AGE diet on AGEs and oxoaldehydes in urine and plasma of abdominally obese individuals

Variable	**Low AGE** (*n*=36)				**High AGE** (*n*=38)				**Low compared to High**	
	**Baseline**	**4 Week**	**Delta**	***p***	**Baseline**	**4 Week**	**Delta**	***p***	**Overall difference***	***p***
**Urine (nmol/mmol creatinin)**										
CML[1]	936.4 ± 221.6	949.1 ± 292.6	12.7 ± 255.2	0.77	980.3 ± 292.6	1101.7 ± 412.0	121.5 ± 399.6	0.07	-124.9 [-276.0,26.2]	0.10
CEL[1]	493.6 ± 138.5	476.9 ± 140.9	-16.8 ± 130.8	0.45	470.5 ± 121.5	762.7 ± 236.0	292.2 ± 260.2	**<0.01**	-286.2 [-376.5,-195.9]	**<0.01**
MG-H1[1]	2343 ± 1196	1902 ± 1061	-441.1 ± 1454	0.08	2399 ± 1218	4452 ± 1958	2053 ± 2286	**<0.01**	-2460 [-3188,-1731]	**<0.01**
Pyrraline[1]	1045 ± 369.6	1267 ± 554.6	222.2 ± 424.2	**<0.01**	991.9 ± 376.7	2142 ± 1028	1151 ± 1105	**<0.01**	-914.4 [-1304,-525.4]	**<0.01**
MGO	128.0 ± 103.1	112.0 ± 40.2	-15.9 ± 79.4	0.24	110.2 ± 46.9	121.6 ± 64.6	11.5 ± 53.4	0.19	-15.2 [-36.8,6.3]	0.16
8-oxo-dG	1.4 ± 0.9	1.4 ± 0.9	0.0 ± 0.6	0.77	1.6 ± 1.4	1.4 ± 1.1	-0.2 ± 1.4	0.48	0.00 [-0.4,0.4]	0.98
CeDG (log)	-1.9 ± 0.8	-2.2 ± 0.6	-0.3 ± 0.7	**0.03**	-1.8 ± 1.9	-1.2 ± 0.	0.7 ± 1.2	**<0.01**	-1.0 [-1.3,-0.6]	**<0.01**
**Plasma (nmol/L)**										
Free CML[1]	87.4 ± 27.7	82.1 ± 27.4	-5.3 ± 31.6	0.32	88.1 ± 35.7	97.0 ± 26.5	8.8 ± 27.4	0.05	-12.5 [-22.8,-2.2]	**0.02**
Free CEL[1]	43.8 ± 11.3	40.2 ± 9.9	-3.6 ± 11.3	0.07	44.3 ± 16.7	81.9 ± 33.7	37.6 ± 31.3	**<0.01**	-39.5 [-50.1,-28.9]	**<0.01**
Free MG-H1[1]	95.7 ± 63.1	77.0 ± 32.0	-18.7 ± 61.9	0.08	105.9 ± 63.1	221.6 ± 122.6	115.7 ± 110.6	**<0.01**	-133.2 [-170.0,-96.4]	**<0.01**
Protein-bound CML[1]	3523 ± 527.7	3444 ± 628	-79.0 ± 387.3	0.24	3525 ± 591.7	3547 ± 666.5	21.5 ± 386.7	0.73	-93.2 [-277.4,91.0]	0.32
Protein-bound CEL[1]	785.5 ± 169.3	764.6 ± 167.5	-20.9 ± 171.2	0.48	790.6 ± 257.6	847.0 ± 263.6	56.4 ± 274.7	0.21	-70.3 [-160.1,19.5]	0.12
Protein-bound MG-H1[1]	1006 ± 269.0	949.9 ± 189.2	-56.4 ± 288.0	0.26	939.3 ± 226.9	969.5 ± 238.0	30.2 ± 192.9	0.34	-45.9 [-139.1,47.3]	0.33
MGO	309.1 ± 42.2	309.6 ± 44.3	-1.8 ± 52.8	0.84	309.1 ± 42.2	307.2 ± 41.9	-7.3 ± 42.5	0.30	0.9 [-17.9,19.7]	0.92
GO	443.0 ± 115.4	400.0 ± 89.6	-43.3 ± 131.8	0.06	445.1 ± 100.9	422.5 ± 100.5	-22.7 ± 101.1	0.18	-18.5 [-60.2,23.2]	0.38
3-DG	1059 ± 96.2	1066 ± 100.4	6.6 ± 65.2	0.55	1114 ± 117.9	1105 ± 79.8	-9.2 ± 65.4	0.39	-2.7 [-27.8,22.5]	0.83
**Skin (arbitrary units)**										
Autofluoresence (SAF)	1.99 ± 0.39	1.96 ± 0.41	-0.03 ± 0.22	0.34	2.03 ± 0.32	2.03 ± 0.38	0.00 ± 0.24	0.98	-0.04 [-0.14,0.07]	0.51

Values are presented as means ± SD. Within-group changes were evaluated with a paired-samples *t* test. Overall differences after the low compared to high AGE diet were evaluated with a one-way ANCOVA with adjustment for age, sex, and the baseline variable of interest. Abbreviations: 3-DG: 3-Deoxyglucose. 8-oxo-dG: 8-Oxo-2'-deoxyguanosine. CEDG: N2-(1-carboxyethyl)-2'-deoxyguanosine. CEL: Nε-(1-carboxyethyl)lysine. CML: Nε-(carboxymethyl)lysine. GO: Glyoxal. MGO: Methylglyoxal. MG-H1: Nδ-(5-hydro-5-methyl-4-imidazolon-2-yl)-ornithine. Pb: Protein-bound.

[1]*n* = 35 for low AGE group due to exclusion of a non-compliant participant.

Supplemental Table 5 Effects of a 4-week low- and high AGE diet on outcomes of glucose metabolism of abdominally obese individuals

Variable	Low AGE (*n*=36)				High AGE (*n*=38)				Low compared to High	
	**Baseline**	**4 Week**	**Delta**	***p***	**Baseline**	**4 Week**	**Delta**	***p***	**Overall difference**	***p***
**Fasting indices**										
Fasting glucose (mmol/L)	4.87 ± 0.44	4.95 ± 0.44	0.08 ± 0.27	**0.08**	5.09 ± 0.49	5.04 ± 0.39	-0.04 ± 0.28	0.36	0.06 [-0.05,0.18]	0.29
Fasting insulin[1] (µIU/ml)	9.50 ± 2.83	9.88 ± 3.50	0.38 ± 2.35	0.34	10.65 ± 5.10	10.34 ± 3.75	-0.31 ± 4.10	0.64	0.17 [-1.19,1.53]	0.80
Fasting c-peptide[1] (pg/L)	1554.4 ± 509.6	1586.3 ± 548.0	31.9 ± 290.3	0.52	1607.9 ± 548.0	1598.1 ± 491.8	-9.8 ± 279.1	0.83	34.7 [-96.5,165.9]	0.60
**Insulin sensitivity**										
Insulin sensitivity[1] (mg/kg/min)	4.53 ± 1.84	4.58 ± 1.91	0.05 ± 1.74	0.86	4.53 ± 1.84	4.66 ± 2.10	0.38 ± 1.46	0.12	-0.25 [-0.96,0.47]	0.49
M/I[1]	4.97 ± 2.57	4.90 ± 2.61	-0.07 ± 2.08	0.85	4.67 ± 2.88	4.91 ± 2.66	0.23 ± 1.59	0.37	-0.20 [-1.01,0.61]	0.63
HOMA (µU·L/mmol·L)	2.09 ± 0.75	2.21 ± 0.84	0.12 ± 0.57	0.23	2.41 ± 1.18	2.33 ± 0.95	-0.08 ± 0.90	0.60	0.07 [-0.24,0.39]	0.65
**Insulin clearance**										
Fasting insulin clearance[1] (L/min/m^2)	1.14 ± 0.19	1.13 ± 0.18	-0.01 ± 0.15	0.69	1.13 ± 0.31	1.10 ± 0.24	-0.03 ± 0.18	0.37	0.02 [-0.04,0.09]	0.49
Steady state insulin clearance[2] (L/min/m^2)	0.49 ± 0.10	0.48 ± 0.09	-0.01 ± 0.06	0.21	0.49 ± 0.10	0.49 ± 0.08	-0.01 ± 0.08	0.54	-0.01 [-0.04,0.02]	0.62
**Insulin secretion**										
Fasting insulin secretion[1] (pmol/min/m^2)	66 ± 20	67 ± 21	1 ± 12	0.58	66 ± 20	66 ± 19	-0 ± 12	0.86	1 [-4,7]	0.66
C-peptide suppression[2] (%)	-13.4 ± 37.7	-6.2 ± 32.1	7.2 ± 36.3	0.26	-9.3 ± 36.8	-1.1 ± 40.1	8.2 ± 28.3	**0.08**	-3.4 [-17.2,10.4]	0.62
Glucose increment $t_{0\text{-}8}$ minutes[3] (mmol/L)	2.79 ± 0.68	2.85 ± 0.62	0.06 ± 0.59	0.56	2.94 ± 0.83	2.66 ± 0.86	-0.29 ± 0.89	**0.06**	0.26 [-0.06,0.58]	0.12
1st phase ISR[3] (pmol/min/m^2)	264 ± 161	285 ± 181	21 ± 68	**0.08**	265 ± 154	267 ± 150	1 ± 77	0.94	17 (-18,51)	0.33
2nd phase ISR[3] (pmol/min/m^2)	150 ± 60	157 ± 73	8 ± 47	0.33	150 ± 50	148 ± 53	-2 ± 37	0.76	11 (-9,30)	0.28
2nd phase ISR steady state[3] (pmol/min/m^2)	179 ± 66	188 ± 92	9 ± 64	0.44	176 ± 61	184 ± 74	8 ± 53	0.35	1 (-26,28)	0.95
β-GS[3] (pmol/min/m^2/mmol·L)	41 ± 19	40 ± 25	-1 ± 19	0.82	39 ± 18	43 ± 24	4 ± 22	0.25	-5 (-15,5)	0.32

Values are presented as means ± SD. Within-group changes were evaluated with a paired-samples *t* test. Overall differences after the low compared to high AGE diet were evaluated with a one-way ANCOVA with adjustment for age, sex, and the baseline variable of interest. Abbrevations: B-GS: beta-cell glucose sensitivity. ISR: Insulin secretion rates. M/I: insulin sensitivity adjusted for plasma insulin.

[1] *n*=35 for low AGE, *n*=38 for high AGE.

[2] *n*=34 for low AGE, *n*=38 for high AGE.

[3] *n*=34 for low AGE, *n*=36 for high AGE.

Supplemental Table 6 Effects of a 4-week low- and high AGE diet on micro and macrovascular function of abdominally obese individuals

Variable	Low AGE (*n*=36)				High AGE (*n*=38)				Low compared to High	
	**Baseline**	**4 Week**	**Delta**	***p***	**Baseline**	**4 Week**	**Delta**	***p***	**Overall difference**	***p***
**Microvascular function**										
IMMR[1] (%)	8.8 ± 41.5	7.6 ± 39.3	1.2 ± 55.4	0.90	5.0 ± 31.6	10.6 ± 28.4	5.5 ± 43.1	0.43	-3.1 [-19.5,13.4]	0.71
Skin heating response (%)	1309.5 ± 645.7	1268.6 ± 733.2	-40.9 ± 821.4	0.77	1444.8 ± 820.6	1251.4 ± 772.7	-193.5 ± 784.5	0.14	33.5 [-292.2,359.1]	0.84
CRAE[2] (µm)	174.4 ± 15.7	173.9 ± 16.1	-0.5 ± 7.9	0.74	174.1 ± 18.7	171.7 ± 17.7	-2.5 ± 6.5	**0.03**	1.9 [-1.7,5.5]	0.30
CRVE[2] (µm)	213.7 ± 13.3	212.3 ± 15.2	-1.4 ± 5.7	0.21	217.0 ± 19.0	212.7 ± 16.7	-4.3 ± 7.3	**<0.01**	1.6 [-2.1,5.2]	0.39
Plasma biomarker of endothelial dysfunction Z-score[3] (SD)	-0.09 ± 1.00	-0.03 ± 1.11	0.06 ± 0.57	0.53	0.09 ± 1.00	0.03 ± 0.89	-0.06 ± 0.52	0.51	0.11 [-0.13,0.35]	0.38
sICAM-1[3] (ng/ml)	350.18 ± 78.66	335.08 ± 77.88	-15.09 ± 44.03	**0.05**	375.09 ± 111.92	361.26 ± 82.96	-13.83 ± 54.69	0.13	-7.50 [-26.65,11.65]	0.44
sVCAM-1[3] (ng/ml)	400.40 ± 94.21	406.49 ± 95.59	6.08 ± 39.37	0.36	391.39 ± 67.99	393.15 ± 61.99	1.76 ± 33.94	0.75	5.77 [-10.62,22.16]	0.49
eSelectin[3] (ng/ml)	83.41 ± 41.54	76.95 ± 38.34	-6.46 ± 13.50	**<0.01**	93.61 ± 48.22	83.09 ± 38.82	-10.52 ± 19.11	**<0.01**	2.52 [-3.24,8.28]	0.39
vWF[3] (%)	103.95 ± 35.56	106.37 ± 32.95	2.42 ± 33.36	0.67	107.21 ± 44.44	100.04 ± 41.25	-7.17 ± 27.67	0.12	9.08 [-3.66,21.82]	0.16
Microvascular flowmotion										
Total power signal (arbitrary units)	88613 ± 95195	127765 ± 142863	39151 ± 133479	0.09	107897 ± 105147	170831 ± 368598	62934 ± 396971	0.34	-39966 [-174475,94542]	0.56
Endothelial contribution (%)	54.7 ± 12.0	53.5 ± 11.0	-1.2 ± 12.7	0.58	52.3 ± 11.3	54.9 ± 9.3	2.6 ± 13.6	0.25	-2.3 [-6.9,2.3]	0.33
Myogenic contribution (%)	9.8 ± 5.6	9.7 ±7.3	-0.1 ± 8.3	0.95	11.3 ± 7.9	10.5 ± 6.5	-0.8 ± 10.3	0.62	-0.6 [-3.9,2.7]	0.71
Neurogenic contribution (%)	31.1 ± 7.9	32.9 ± 7.1	1.8 ± 7.1	0.14	31.9 ± 8.2	30.8 ± 6.5	-1.1 ± 7.7	0.37	2.7 [-0.0;5.4]	0.05
Cardiogenic contribution (%)	2.2 ± 2.8	1.8 ± 2.3	-0.4 ± 3.2	0.42	1.8 ± 1.9	1.9 ± 2.1	0.1 ± 1.6	0.79	-0.2 [-1.2,0.7]	0.64
Respiratory contribution (%)	2.1 ± 2.0	2.1 ± 1.7	-0.1 ± 2.6	0.88	2.6 ± 2.9	1.9 ± 2.0	-0.7 ± 1.6	0.07	0.4 [-0.4,1.2]	0.31
**Macrovascular function**										
FMD[3] (%)	3.5 ± 2.9	3.5 ± 3.0	-0.0 ± 2.2	0.99	3.4 ± 2.8	3.6 ± 3.2	0.16 ± 4.5	0.82	-0.1 [-1.5,1.2]	0.85
cfPWV[3] (m/s)	10.2 ± 2.5	9.7 ± 1.7	-0.6 ± 1.9	**0.08**	9.9 ± 2.8	10.0 ± 2.7	0.1 ± 1.1	0.76	-0.4 [-1.0,0.2]	0.24
Carotid DC[3] (10^3/kPa)	18.1 ± 9.0	17.8 ± 7.9	-0.3 ± 5.8	0.77	17.4 ± 9.5	16.8 ± 8.4	-0.5 ± 4.5	0.48	0.0 [-2.0,2.0]	0.98
Carotid YEM[3] (10^3kPa)	0.65 ± 0.29	0.63 ± 0.27	-0.02 ± 0.21	0.62	0.64 ± 0.33	0.64 ± 0.27	-0.00 ± 0.20	0.95	0.01 [-0.07,0.09]	0.87
Carotid IMT[3] (um)	787 ± 117	806 ± 145	18 ± 78	0.17	839 ± 137	825 ± 148	14 ± 119	0.46	21 [-24,67]	0.36
Aix[1] (%)	20.8 ± 13.6	21.7 ± 12.6	0.9 ± 0.8	0.26	21.6 ± 10.2	22.0 ± 11.3	0.4 ± 6.6	0.74	0.8 [-1.6,3.2]	0.50
24-h systolic BP[4] (mmHg)	126.5 ± 11.8	125.8 ± 12.0	-0.7 ± 7.4	0.59	123.7 ± 8.8	125.2 ± 10.2	1.6 ± 6.6	0.17	-1.9 [-5.3,1.6]	0.28
24-h diastolic BP[4] (mmHg)	80.4 ± 8.1	79.7 ± 8.8	-0.7 ± 5.0	0.41	80.4 ±8.1	79.7 ± 8.8	0.2 ± 4.7	0.83	-0.2 [-2.5,2.2]	0.87

Values are presented as means ± SD. Within-group changes were evaluated with a paired-samples *t* test. Overall differences after the low compared to high AGE diet were evaluated with a one-way ANCOVA with adjustment for age, sex, and the baseline variable of interest. Abbreviations: Aix: Augmentation index. bp: blood pressure. Carotid DC: Carotid Distensibility Coefficient. Carotid YEM: Carotid Young's Elastic Modulus. cfPWV: carotid-femoral Pulse Wave Velocity. CRAE and CRVE: Central retinal arteriolar and venular equivalent. FMD: flow mediated dilation. sICAM-1: soluble intracellular adhesion molecule-1. sVCAM-1: soluble vascular adhesion molecule-1. vWF: von Willebrand factor.

[1] *n*=34 for low AGE, *n*=38 for high AGE.

[2] *n*=30 for low AGE, *n*=37 for high AGE.

[3] *n*=35 for low AGE, *n*=38 for high AGE.

[4] *n*=32 for low AGE, *n*=36 for high AGE.

Supplemental Table 7 Effects of a 4-week low- and high AGE diet on inflammatory markers and leukocyte differentiation of abdominally obese individuals

Variable	Low AGE (*n*=35)				High AGE (*n*=38)				Low compared to High	
	**Baseline**	**4 Week**	**Delta**	***p***	**Baseline**	**4 Week**	**Delta**	***p***	**Overall difference**	***p***
**Inflammatory markers**										
Plasma inflammatory markers Z-score (SD)	-0.02 ± 0.95	0.07 ± 1.07	0.09 ± 0.55	0.31	0.02 ± 1.06	-0.07 ± 0.93	0.09 ± 0.58	0.35	0.18 [-0.08,0.44]	0.17
Adiponectin (ug/ml)	14.86 ± 5.44	13.52 ± 4.65	-1.34 ± 1.61	**<0.01**	15.00 ± 5.50	15.17 ± 6.04	0.19 ± 1.91	0.55	-1.54 [-2.37,-0.71]	**<0.01**
MCP-1 (pg/ml)	103.49 ± 27.90	98.53 ± 23.41	-4.95 ± 17.3	0.10	101.45 ± 18.27	99.32 ± 18.12	-2.13 ± 11.3	0.25	-1.01 [-6.89,4.87]	0.73
IL-6 (log)	-0.31 ± 0.65	-0.35 ± 0.56	-0.04 ± 0.43	0.55	-0.31 ± 0.54	-0.43 ± 0.53	-0.11 ± 0.40	0.09	0.07 [-0.11,0.24]	0.46
IL-8 (pg/ml)	3.60 ± 2.92	3.92 ± 3.89	0.32 ± 1.28	0.14	3.35 ± 1.15	3.21 ± 0.84	-0.15 ± 0.87	0.31	0.36 [-0.11,0.82]	0.13
TNFα (pg/ml)	1.16 ± 0.30	1.17 ± 0.28	0.01 ± 0.14	0.64	1.19 ± 0.26	1.16 ± 0.18	-0.04 ± 0.13	0.10	0.04 [-0.02,0.09]	0.16
CRP (log)	0.91 ± 0.92	0.56 ± 0.90	-0.35 ± 0.70	**<0.01**	0.72 ± 1.27	0.39 ± 1.02	-0.33 ± 0.70	**<0.01**	0.04 [-0.25,0.33]	0.77
SAA (µg/ml)	8.67 ± 10.84	5.92 ± 4.54	-2.75 ± 11.21	0.15	13.71 ± 42.63	5.51 ± 4.37	-8.20 ± 39.30	0.21	0.78 [-0.84,2.40]	0.34
sICAM-1 (ng/ml)	350.18 ± 78.66	335.08 ± 77.88	-15.09 ± 44.03	**0.05**	375.09 ± 111.92	361.26 ± 82.96	-13.83 ± 54.69	0.13	-7.50 [-26.65,11.65]	0.44
**Leukocyte differentiation**										
Leucocytes (10^9/L)	5.92 ± 1.25	5.70 ± 1.16	-0.21 ± 0.66	**0.06**	6.07 ± 1.47	5.73 ± 1.38	-0.34 ± 0.65	**<0.01**	0.10 [-0.19,0.39]	0.49
Segm. Granulocytes (10^9/L)	3.49 ± 1.00	3.28 ± 0.95	-0.21 ± 0.63	**0.05**	3.63 ± 1.08	3.38 ± 1.02	-0.21 ± 0.63	**0.01**	0.02 [-0.25,0.28]	0.89
Lymphocytes (10^9/L)	1.80 ± 0.49	1.80 ± 0.47	-0.00 ± 0.24	0.99	1.79 ± 0.49	1.74 ± 0.45	-0.05 ± 0.21	0.15	0.04 [-0.05,0.14]	0.38
Monocytes (10^9/L)	0.48 ± 0.12	0.47 ± 0.10	-0.00 ± 0.08	0.82	0.48 ± 0.17	0.43 ± 0.13	-0.05 ± 0.10	**<0.01**	0.05 [0.01,0.08]	**0.01**
Eosinophils (10^9/L)	0.12 ± 0.05	0.13 ± 0.07	0.01 ± 0.05	0.46	0.15 ± 0.11	0.15 ± 0.09	0.00 ± 0.08	0.96	-0.00 [-0.03,0.03]	0.83
Basophils (10^9/L)	0.05 ± 0.03	0.05 ± 0.02	0.01 ± 0.02	**0.09**	0.04 ± 0.03	0.05 ± 0.02	0.01	0.12	0.00 [-0.01,0.01]	0.94

Values are presented as means ± SD. Within-group changes were evaluated with a paired-samples *t* test. Overall differences after the low compared to high AGE diet were evaluated with a one-way ANCOVA with adjustment for age, sex, and the baseline variable of interest. Abbreviations: CRP: C-reactive Protein. MCP-1: IL-6: Interleukin-6, IL-8: Interleukin-8. Monocyte Chemoattractant Protein-1. SAA: Serum Amyloid A. Segm: Segmented. sICAM-1: soluble intracellular adhesion molecule-1. TNFα: Tumor Necrosis Factor alpha.

Supplemental Table 8 Effects of a 4-week low- and high AGE diet on liver-associated outcomes of abdominally obese individuals

Variable	Low AGE (*n*=35)				High AGE (*n*=38)				Low compared to High	
	**Baseline**	**4 Week**	**Delta**	***p***	**Baseline**	**4 Week**	**Delta**	***p***	**Overall difference**	***p***
Gamma-GT (log)	3.00 ± 0.51	2.99 ± 0.49	-0.02 ± 0.19	0.62	3.13 ± 0.61	3.03 ± 0.58	-0.10 ± 0.26	**0.03**	0.07 [-0.04,0.17]	0.19
Triglycerides (mmol/L)	1.14 ± 0.43	1.17 ± 0.40	0.03 ± 0.29	0.51	1.59 ± 0.79	1.49 ± 0.80	-0.11 ± 0.31	**0.05**	0.09 [-0.05,0.24]	0.21
LDL-cholesterol (mmol/L)	3.30 ± 0.91	3.22 ± 0.94	-0.08 ± 0.44	0.27	3.72 ± 0.75	3.59 ± 0.91	-0.13 ± 0.49	0.11	0.03 [-0.19,0.25]	0.76
HDL-cholesterol (mmol/L)	1.47 ± 0.41	1.41 ± 0.36	-0.06 ± 0.15	**0.02**	1.30 ± 0.29	1.32 ± 0.32	0.02 ± 0.15	0.38	-0.06 [-0.13,0.01]	0.09
Fatty liver index (unitless)	57.35 ± 22.66	55.61 ± 23.69	-1.75 ± 7.17	0.15	63.63 ± 20.58	60.62 ± 21.83	-3.01 ± 8.14	**0.03**	1.37 [-2.30,5.03]	0.46

Values are presented as means ± SD. Within-group changes were evaluated with a paired-samples *t* test. Overall differences after the low compared to high AGE diet were evaluated with a one-way ANCOVA with adjustment for age, sex, and the baseline variable of interest. Abbreviations: Gamma-GT: Gamma-glutamyltransferase.

Supplemental Table 9 Multivariate-adjusted associations between indices of AGE intake and outcomes in 72 abdominally obese individuals after a low and high AGE diet.

**AGEs**	**Plasma Adiponectin**	**Plasma monocyte count**	**Urinary CeDG**	**cfPWV**	**Serum HDL**	**Serum Triglycerides**
SD	mmol/L	10^9/L	nmol/mmol kreat	m/s	mmol/L	mmol/L
**Diet**						
CML	**0.85 [0.26,1.44]**	-0.02 [-0.04,0.00]	**0.11 [0.03,0.19]**	0.23 [-0.18,0.64]	**0.05 [0.00,0.10]**	**-0.12 [-0.21,-0.02]**
CEL	**0.82 [0.24,1.39]**	-0.02 [-0.04,0.00]	**0.12 [0.04,0.19]**	0.29 [-0.11,0.69]	**0.05 [0.00,0.09]**	**-0.12 [-0.21,-0.02]**
MG-H1	**0.94 [0.32,1.55]**	-0.02 [-0.05,0.00]	**0.11 [0.02,0.19]**	0.24 [-0.20,0.67]	**0.06 [0.01,0.11]**	**-0.12 [-0.22,-0.01]**
**Urine**						
CML	**-0.48 [-0.95,-0.02]**	0.00 [-0.01,0.02]	**0.09 [0.03,0.15]**	-0.10 [-0.42,0.22]	-0.01 [-0.05,0.02]	-0.05 [-0.12,0.03]
CEL	0.27 [-0.24,0.78]	-0.01 [-0.03,0.01]	**0.13 [0.07,0.19]**	0.04 [-0.30,0.38]	-0.01 [-0.05,0.03]	**-0.09 [-0.17,-0.02]**
MG-H1	0.08 [-0.44,0.59]	-0.01 [-0.03,0.01]	**0.18 [0.13,0.23]**	-0.11 [-0.45,0.23]	-0.02 [-0.06,0.03]	**-0.08 [-0.16,-0.00]**
Pyrraline	0.16 [-0.34,0.66]	-0.01 [-0.03,0.01]	**0.12 [0.06,0.18]**	0.10 [-0.24,0.44]	-0.02 [-0.06,0.02]	-0.07 [-0.15,0.01]
**Plasma**						
CML	-0.37 [-0.91,0.16]	-0.02 [-0.04,0.00]	**0.13 [0.07,0.20]**	-0.06 [-0.42,0.31]	0.01 [-0.04,0.05]	0.01 [-0.08,0.09]
CEL	0.11 [-0.42,0.63]	-0.01 [-0.03,0.01]	**0.11 [0.04,0.17]**	-0.13 [-0.48,0.22]	-0.00 [-0.04,0.04]	**-0.12 [-0.20,-0.05]**
MG-H1	0.04 [-0.50,0.57]	-0.01 [-0.03,0.01]	**0.17 [0.11,0.23]**	-0.22 [-0.59,0.15]	-0.01 [-0.05,0.03]	**-0.12 [-0.20,-0.04]**

Beta's (B) and 95% CIs indicate the difference in outcome per unit change in determinant. Please note that AGEs in urine and plasma were standardized. Associations were adjusted for age, sex, and intake of carbohydrates, fat, and protein as energy-percentages. Statistically significant associations are shown bold.
